# Symptom Clusters and Longitudinal Progression in Chronic Hemodialysis Patients: A Prospective Single-Center Study

**DOI:** 10.3390/healthcare14101375

**Published:** 2026-05-18

**Authors:** Naama Altura, Gillie Gabay, Ruth Israeli, Baher Usman, Safa Abu Lail, Rely Alon, Iddo Z. Ben-Dov, Revital Zelker

**Affiliations:** 1 Dialysis Unit “Ziv”, Hadassah Ein-Kerem Medical Center, Jerusalem 91120, Israel; 2 School of Science, Achva Academic College, Shikmim 79804, Israel; gillieg@live.achva.ac.il; 3 Nursing and Health Professions, Hadassah Medical Center, Jerusalem 91120, Israel; 4 Department of Nephrology and Hypertension, Hadassah Medical Organization, Jerusalem 91120, Israel; iddo@hadassah.org.il; 5 Faculty of Medicine, Hebrew University of Jerusalem, Jerusalem 91904, Israel; 6 Hadassah Research and Innovation Center in Nursing, Hadassah Ein-Kerem Medical Center, Jerusalem 91120, Israel

**Keywords:** chronic kidney disease, burden of symptoms, clinical nurse leadership, cluster-based symptomology, distress, fatigue, gastrointestinal, hemodialysis patients, perceived health, temporal instability

## Abstract

**Highlights:**

**What are the main findings?**

- Symptom clusters exhibit significant temporal instability, reflecting a dynamic reorganization of the patient experience over a 12-month period.
- While fatigue and sleep disturbances remain persistently high-burden, gastrointestinal symptoms show a significant longitudinal improvement.

**What are the implications of the main findings?**

- Clinical assessment may benefit from a greater emphasis on longitudinal monitoring rather than solely cross-sectional evaluations, to capture the evolving nature of symptom clusters.
- Vascular access type (CVC) and Diabetes Mellitus emerged as important patient characteristics associated with symptom burden and may inform more personalized symptom management approaches in future studies.

**Abstract:**

**Background**: Chronic hemodialysis (HD) patients face symptoms that significantly impact their quality of life and health outcomes. Longitudinal research on the dynamics of symptom severity and the integration of individual patient characteristics into cluster analyses is limited, hindering understanding of cluster evolution over time. **Objective**: The objective of this study was to characterize and compare symptom clusters across body systems based on frequency and severity at three time points in chronic HD patients. **Methods**: This prospective longitudinal study collected self-reported data on 23 symptoms using validated measures from 69 chronic HD patients (age range: 24–87 years) at three time points over a year. Symptoms were rated on a 0–10 scale. Symptom progression and clustering were analyzed using heat maps and principal component analysis. **Results**: Among 69 HD patients, a substantial symptom burden was identified at baseline, with fatigue, overall perceived health, worry or distress, and sleep disturbance reported as the most severe (mean scores > 4.0 on a 0–10 scale). Hierarchical clustering yielded a five-cluster solution; however, longitudinal analysis revealed poor structural stability in patient symptom profiles over 12 months (ARI < 0.70), indicating significant symptomatic reorganization. Gastrointestinal cluster showed a statistically significant reduction in severity over time (β = −0.914, *p* = 0.003); fatigue and overall perceived health remained a high burden. Subgroup analyses demonstrated that patients using central venous catheters reported significantly higher severity in pain, fatigue, and nausea compared to patients with arteriovenous fistulas, while Diabetes mellitus was uniquely associated with increased dyspnea (*p* < 0.001). **Conclusions**: Chronic HD patients experience a dynamic and multidimensional symptom burden, with significant variations in severity, progression, and clustering of symptoms over time. The observed temporal instability of symptom clusters and the heterogeneity of individual trajectories emphasize the importance of routine, longitudinal symptom assessment and flexible, patient-centered management strategies by nephrology nurse specialists, which may support value-based healthcare approaches.

## 1. Introduction

HD (HD) is the most common form of kidney replacement therapy worldwide, accounting for approximately 69% of all kidney replacement therapy and 89% of all dialysis [1]. The global burden of the disease is gradually increasing [2,3,4]. Despite significant advances in dialysis technology, patients undergoing maintenance HD carry a high burden of physical, emotional, and cognitive symptoms that profoundly impair their quality of life (QoL) and functional capacity [5].

A landmark global systematic review and meta-analysis of 449 studies encompassing almost 200 thousand chronic kidney disease (CKD) patients across 62 countries reported that the most prevalent symptoms in dialysis populations were lack of energy (66%), poor mobility (58%), pain (65%), and sleep disturbance, with a median of seven concurrent symptoms per patient and nearly half of all patients rating at least one symptom as severe or overwhelming [5]. Longitudinal data further confirm this burden: patients routinely report between nine and twelve concurrent symptoms across successive assessments, with fatigue, dry skin, and worrying emerging as among the most prevalent [6].

High symptom scores are consistently associated with reduced physical and mental health-related QoL, greater perceived disease burden, and worse functional capacity [7]. Dialysis patients’ symptom profiles are shaped not only by uremia and dialysis-related physiological changes, but also by individual characteristics such as age, diabetes mellitus (DM) status, vascular access type, and clinical laboratory parameters [8,9]. These observations underscore the need to move beyond disease-level summaries toward individualized, patient-centered approaches to symptom assessment and disease management.

CKD is a silent and expensive condition, as by the time symptoms appear, the damage is often irreversible, making HD one of the most resource-intensive treatments in medicine. As hospitals move from fee-for-service to value-based healthcare (VBHC), the goal is to increase quality and improve clinical outcomes while reducing overall costs of care [10,11]. Applying VBHC in HD enables the detection of CKD earlier than in the emergency room, improving disease trajectory, reducing infections, and enhancing patient experience and QoL [12].

Over the past two decades, the CKD field has shifted from studying isolated symptoms to recognizing that symptoms occur in groupings, termed symptom clusters that share underlying biological mechanisms and may respond collectively to interventions [8]. The clinical relevance of this shift is substantial: By focusing on symptom clusters rather than isolated symptoms, clinicians may be better positioned to identify patients at higher risk, support earlier recognition of symptom escalation, and inform the development of more targeted symptom management strategies, with potential implications for quality of life and healthcare resource utilization [6,12,13].

Multiple studies have converged on a consistent set of symptom clusters across diverse HD populations and settings. Gastrointestinal symptoms (nausea, vomiting, decreased appetite, diarrhea, and constipation), sleep-disturbance symptoms (trouble falling or staying asleep, drowsiness, and fatigue), dermatological symptoms (dry skin, pruritus, and dry mouth), and emotional or psychological symptoms (worry, anxiety, sadness, and irritability) each recur across studies [6,13,14,15]. A recent systematic review of eight studies confirmed this consistency, identifying gastrointestinal, neuromuscular, dermatological, and emotional symptom clusters as the most reproducible, while noting that some uremic symptoms, such as shortness of breath, numbness, and vomiting, often form a distinct group [14].

The clinical impact of symptom clusters on patient outcomes is well established. Clusters related to lack of energy and reduced mobility consistently predict difficulty performing daily activities across CKD populations, including HD patients [7]. Emotional clusters (e.g., anxiety and depression) are strong predictors of reduced self-efficacy and perceived disease burden. Furthermore, mobility-related and pain clusters further diminish functional capacity and overall QoL [9]. Higher uremic and gastrointestinal cluster scores are associated with poorer CKD-specific QoL scores and lower Karnofsky Performance Status, indicating that untreated symptoms are functionally disabling [6].

Longitudinal data on symptom cluster evolution remain scarce [14]. The few available longitudinal studies suggest that while the cluster structure (uremic, gastrointestinal, skin, and emotional groupings) tends to persist, individual symptoms frequently migrate between clusters across assessments. In a one-year longitudinal study of 271 dialysis patients, decreased appetite, headache, and muscle soreness shifted cluster membership across different time points; the skin cluster demonstrated the greatest stability, whereas the uremic cluster was most variable [6]. These findings highlight the dynamic nature of symptom experience and underscore the inadequacy of single-time point assessments for guiding clinical management.

### Rationale and Aims of the Present Study

Despite growing interest in symptom clusters among HD patients, two critical gaps persist: the predominance of cross-sectional designs that preclude understanding of cluster evolution over time, and the limited integration of individual patient characteristics into cluster analyses. Researchers called for more accurate and cost-effective diagnostic tools and interventions [2,3,4]. In response to these calls, the present nurse-led study directly addresses these gaps. This study prospectively assessed 23 self-reported symptoms plus general perceived health across three time points spanning one year in 69 chronic HD patients treated at a single ambulatory dialysis unit. Using heat map visualization and Principal Component Analysis (PCA), we characterized the structure and progression of symptom clusters and examined how cluster patterns varied according to patient characteristics. This single-center study was designed as a descriptive, hypothesis-generating investigation, aiming to characterize symptom clusters and their longitudinal trajectories rather than to generate population-level estimates or to evaluate intervention effectiveness. By capturing the dynamic, multidimensional nature of symptom experience in a real-world, longitudinally observed rare cohort, this study aims to provide a more clinically nuanced understanding of symptom burden in chronic HD, which may support the development of tailored, nurse-led symptom management strategies. This proof-of-concept study demonstrates nurses’ capacity to care for high-risk HD patients during the current shortage of nephrologists, thereby promoting VBHC.

## 2. Materials and Methods

This prospective, longitudinal, single-center observational study was conducted in the ambulatory HD unit at a tertiary Israeli medical center. The institutional Helsinki Committee granted Ethical approval (Helsinki Committee approval # 20-0147). A waiver of informed consent was granted because symptom data were collected solely through anonymized self-report questionnaires, with no linkage to clinical records or patient identifiers. Therefore, no laboratory or medical record data were accessed or collected.

Adult patients (≥18 years) receiving maintenance HD three times weekly for at least 3 months before enrolment were eligible for inclusion. Exclusion criteria were acute hospitalization in the preceding month, active Hematologic-Oncologic disease, inability to communicate in Hebrew or Arabic, and cognitive impairment precluding completion of the questionnaire. A consecutive sample of 69 patients was enrolled between 4 October 2022 and 6 December 2024. This rare cohort represents a convenience sample recruited based on feasibility considerations inherent to prospective longitudinal research in a clinically vulnerable population.

During the study period, patients received standard maintenance HD care according to routine clinical practice at the study center, including thrice-weekly dialysis sessions and regular medical and nursing follow-up. No study-specific interventions or changes to routine care were introduced; thus, observed symptom patterns reflect real-world experience under usual care conditions.

Symptom severity was assessed using two questionnaires. First, the MDASI questionnaire (MD Anderson Symptom Inventory) [16] which includes 13 symptoms (pain, fatigue, nausea, sleep disturbance, distress/feeling upset, shortness of breath, difficulty remembering, decreased appetite, drowsiness, sadness, dry mouth, vomiting, and numbness/tingling). The MDASI has several advantages over other symptom-assessment scales. The MDASI assesses both symptom severity and symptom interference with daily life, making it applicable across cancer types and treatments, and can be adapted to specific cancer types and treatments. Also, a numerical scale ranging from 0 (absent) to 10 (worst imaginable) is easy for patients speaking different languages to understand and complete.

Second, based on the Dialysis Symptom Index (DSI) [17], a set of ten core symptoms, including constipation, diarrhea, muscle cramps, leg edema, inattention, restless legs, cough, poor concentration, dry skin, and pruritus. To balance clinical relevance and feasibility in routine care, the MDASI was supplemented with selected dialysis-specific symptoms from the DSI rather than administering the full index.

The final module of symptoms and their severity comprised 23 items. The questionnaire underwent a forward-backward translation process for linguistic validation in both Hebrew and Arabic, conducted by two independent translators to ensure conceptual equivalence and cultural adaptation of the instrument.

To assess perceived health status, a single item was used [18]. Despite its simplicity, this measure has been validated in both research and clinical settings and has demonstrated predictive value for mortality [19]. Patients are asked to rate their overall health on a 4-point scale ranging from 1 (good) to 4 (very poor).

Clinical parameters included the presence of diabetes mellitus (DM), dialysis access type (arteriovenous fistula vs. central venous catheter), dialysis vintage, and treatment frequency. Demographic characteristics included age, gender, religion, religiosity, and level of education. Socioeconomic status was calculated based on household density (number of individuals per room) and occupational status. The questionnaire was administered by trained nursing staff in Hebrew or Arabic according to patient preference. Data were collected at three time points with four to six months intervals between measurements: baseline (T1, [Month 0]), mid-study (T2, [Month 6]), and end of study (T3, [Month 12]). Assessments were performed during routine dialysis sessions. Notably, T1 represents baseline at study enrollment (not necessarily dialysis initiation), as participants were at varying dialysis vintage. Because symptom assessments were conducted when patients were available for participation, not all enrolled patients completed questionnaires at each assessment. Consequently, the number of completed observations varied across T1, T2, and T3.

### Data Analysis

Patient characteristics and symptom severity scores are reported as mean ± standard deviation (SD) for continuous variables and frequency (%) for categorical variables. Symptom severity at each time point was compared using paired *t*-tests/Wilcoxon signed-rank tests as appropriate for data distribution.

To identify groups of co-occurring symptoms, hierarchical agglomerative clustering was performed on a pooled symptom-by-patient severity matrix, focusing on relationships among symptoms rather than clustering patients. Ward’s linkage method and Canberra distance were used, as this combination is robust to differences in scale and variability. This approach was chosen to identify symptom co-occurrence patterns across the cohort. The optimal number of clusters was determined using the within-cluster sum of squares (“elbow method”). Cluster solutions were visualized using annotated heat maps generated with the heat map package in R (version 4.5.2). The stability of the identified clusters was assessed by repeating the analysis separately for T1, T2, and T3. We used linear mixed-effects models, which account for within-subject correlation and allow inclusion of participants with incomplete follow-up using all available observations. Cluster solution stability was further evaluated by computing the average silhouette width (ASW) using Canberra distance.

PCA was applied to the symptom severity matrix to identify dominant axes of symptom variation. Components with eigenvalues >1 were retained. PCA was applied as an independent visualization check to assess whether symptoms within each hierarchical cluster co-localize; it was not used in cluster derivation. To visualize inter-symptom similarity across patients, a symptom-space dimensionality reduction approach was applied based on the Generalized Dissimilarity Metric 2 (GDM2; ClusterSim package clusterSim_0.51-6). A symptom × patient matrix was submitted to GDM2 to compute a pairwise symptom dissimilarity matrix, which was then projected to two dimensions by classical multidimensional scaling (cmdscale, R stats package). The resulting map was visualized with symptoms color-coded by a priori cluster assignment (gastrointestinal, neuropathic, neurological, respiratory, psychological, skin/mucous membranes). This analysis was performed separately for T1, T2, T3, and for per-patient mean scores across all time points.

Symptom progression over time was characterized by computing the mean change in severity between time points for each symptom and visualized using a longitudinal heat map. To formally assess symptom trajectories, linear mixed-effects models were fitted for each symptom cluster, with mean cluster severity as the outcome, time (in years from baseline) as a fixed effect, and patient as a random intercept, using the lme4 package in R. Fixed-effect estimates (β coefficients) with 95% confidence intervals and *p*-values are reported. As a sensitivity analysis, diabetes mellitus (DM) status was added as a fixed effect to each model (cluster score ~ time + DM + [1|patient]) to assess whether the primary time-effect estimates were robust to adjustment for this covariate. DM was the single variable that showed a statistically significant association with symptom burden in the cross-sectional subgroup analysis.

Differences in symptom profiles between subgroups (sex, diabetes mellitus status, dialysis access type, and age group [≥65 vs. <65 years]) were assessed using the Mann–Whitney U test/independent *t*-test for individual symptoms and MANOVA for the overall symptom profile. All tests were two-tailed, and a significance threshold of *p* < 0.05 was applied. Multiplicity in subgroup analyses was controlled using the Benjamini–Hochberg false discovery rate correction. Statistical analyses were performed in R (version 4.5.2).

Associations between symptom severity and sociodemographic or treatment-related variables were examined using per-patient mean symptom severity scores averaged across all three time points. For continuous predictors (dialysis vintage in months; household density expressed as persons per room), Spearman rank correlations were computed. For ordinal predictors (treatment frequency per week, religiosity, education level, occupational status, and financial satisfaction), Kendall’s τb was used, as it more appropriately accounts for tied ranks inherent in Likert-type scales. Religion (nominal categorical) was analyzed with the Kruskal–Wallis test. All *p*-values were adjusted for multiple comparisons using the Benjamini–Hochberg false discovery rate procedure applied across all symptom–variable pairs. Results are presented as a heat map of correlation coefficients (Spearman ρ or Kendall τb, as applicable), and as a tabulated summary.

Artificial intelligence–assisted code generation (Claude version 1.7196.0; Anthropic, San Francisco, CA, USA) was used to support data visualization and script development. All statistical analyses were performed using established R packages (lme4 (version 2.0-1), lmerTest (version 3.2-1), cluster (version 2.1.8.2), and pheatmap (version 1.0.13)). No AI tool was used to perform or interpret statistical analyses autonomously with all outputs manually verified for accuracy and clinical relevance by the research team.

## 3. Results

A total of 69 patients undergoing maintenance HD were enrolled. The cohort had a mean age of 63.5 ± 15.7 years (range 24–87 years). The majority were male (n = 48, 69.6%), with females comprising 21 participants (30.4%) and nearly half had diabetes mellitus (n = 34, 47.9%). HD was delivered predominantly via arteriovenous fistula (n = 50, 72.4%), with the remainder accessing treatment through a central venous catheter (n = 19, 27.5%). A minority of patients had undergone prior kidney transplantation (n = 11, 15.5%) (Table 1).

Symptom questionnaires were administered at three time points: baseline (T1), approximately six months (T2), and approximately 12 months (T3) after enrolment. Due to rolling recruitment and health-related attrition, not all participants completed assessments at all time points.

A total of 45 participants completed the baseline assessment (T1), 42 completed the six-month assessment (T2), and 31 completed the 12-month assessment (T3). Of these, 40 participants completed both T1 and T2, 30 completed both T2 and T3, and 29 participants completed all three assessments. Attrition during follow-up was primarily attributable to clinical deterioration, kidney transplantation, or death.

At baseline, patients reported substantial symptom burden across multiple domains. The highest mean severity scores were observed for fatigue (5.13 ± 3.08), overall perceived health (4.83 ± 1.35), worry or distress (4.18 ± 3.22), and sleep disturbance (4.11 ± 3.11), each exceeding 4.0 on the 0–10 scale. In contrast, leg edema (2.07 ± 2.69) and vomiting (1.64 ± 2.21) were the least severe symptoms at baseline.

At approximately six months (T2), most symptoms showed a reduction in mean severity. By 12 months (T3), fatigue returned to levels comparable to baseline (5.33 ± 3.37), whereas nausea (0.67 ± 1.62) and vomiting (0.83 ± 2.20) showed marked and sustained improvement. Overall perceived health remained a high-burden symptom across all three time points (T1: 4.83, T2: 4.53, T3: 4.28). Mean severity scores for all symptoms at each time point are presented in Table 2.

**Figure 1:**
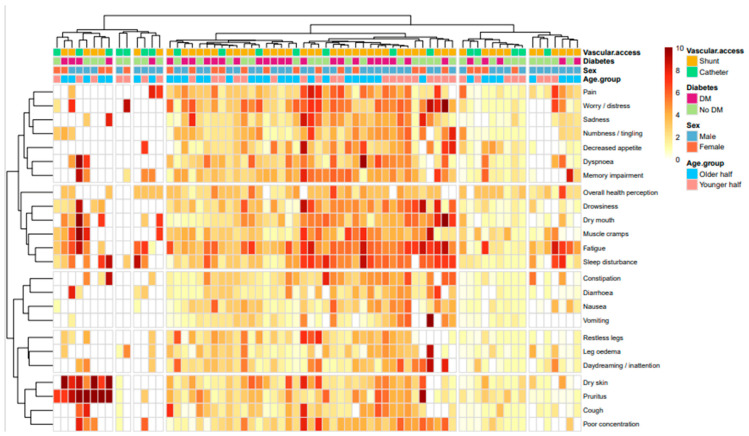
Annotated heat map of symptom severity (24 symptoms × patients; Ward D2 + Canberra distance). Five symptom clusters shown as row dendrograms; four patient-level annotation tracks (age, sex, diabetes, vascular access).

Hierarchical clustering was applied to the pooled symptom-score matrix using Ward’s D2 linkage and Canberra distance, which is robust to the sparse and skewed distributions typical of symptom data. Inspection of the within-cluster sum-of-squares (elbow) plot indicated a natural inflection at k = 5 clusters (Figure 1; Supplementary Figure S1—Cluster Elbow Plot), which was therefore selected as the final solution. The average silhouette width was maximized at k = 5 (ASW = 0.08; Supplementary Figure S6), indicating weak separation between clusters; accordingly, the clustering solution should be interpreted as exploratory.

This five-cluster solution was further supported by clinical interpretability, as described below in Table 3.

**Figure 2:**
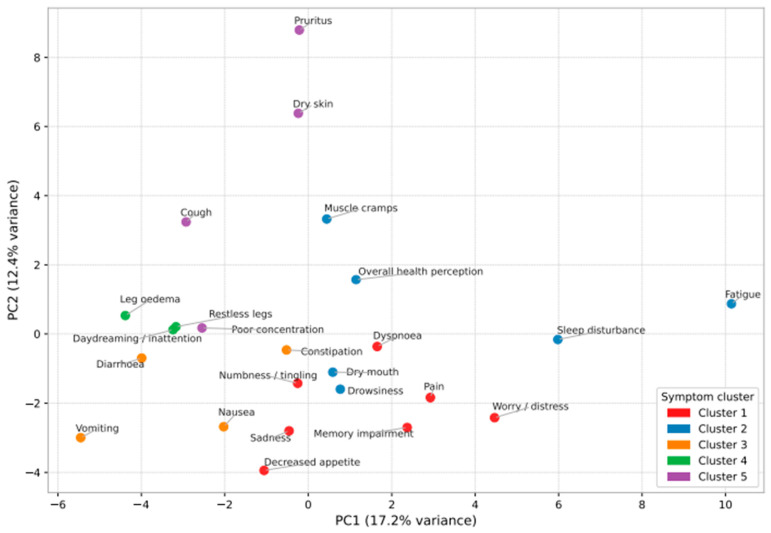
PCA plot of symptom profiles. Each symptom is plotted in the space of principal components 1 and 2 and color-coded by cluster (k = 5).

The five-cluster solution is visualized as an annotated heat map in Figure 1 and as a principal component analysis (PCA) plot in Figure 2. Patient-level annotation tracks (age group, sex, diabetes status, and vascular access type) are overlaid on the heat map to allow visual inspection of potential demographic patterning within clusters.

The temporal stability of the five-cluster symptom structure was evaluated using the Adjusted Rand Index (ARI), which quantifies agreement between clustering solutions applied independently at each time point (ARI = 1.0 indicates perfect agreement; ARI = 0 indicates chance-level agreement, negative values suggest poorer-than-random clustering). Cluster agreement between baseline and six months was moderate (ARI = 0.588). In contrast, agreement between baseline and 12 months (ARI = 0.023), and agreement between six and 12 months (ARI = −0.013) was absent. All comparisons fell below the commonly accepted threshold for acceptable stability (ARI > 0.70), indicating that patient symptom profiles reorganized substantially over the study period.

At baseline, the mean number of symptoms per patient rated as clinically significant was 10.15 (SD 6.92, median 9.5); this decreased to 5.93 (SD 5.48, median 5.5) at six months, before rising to 8.69 (SD 5.57, median 8.0) at 12 months. The most prevalent clinically significant symptom at baseline was overall perceived health (80.0%), followed by fatigue (75.0%), sleep disturbance (60.0%), and worry or distress (60.0%).

**Figure 3:**
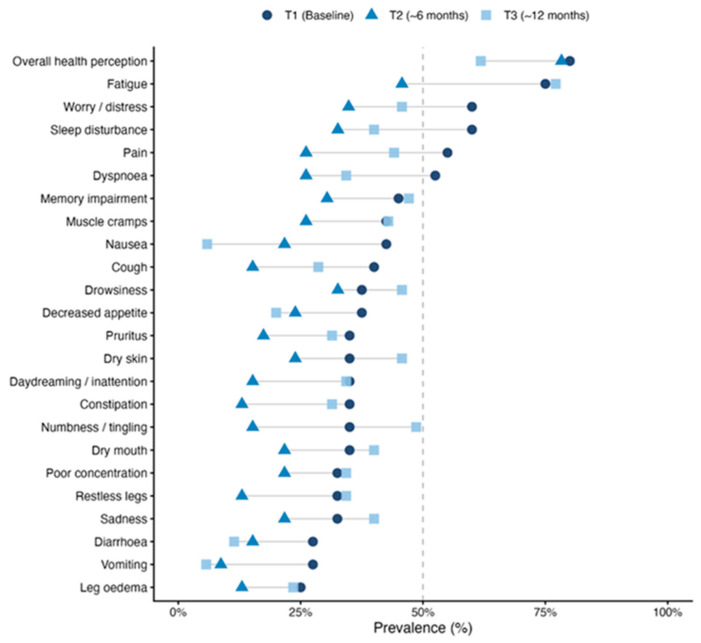
Dot plot of clinically significant symptom prevalence (%), sorted by baseline (T1) prevalence (severity rating ≥ 4 on the 0–10).

Notable longitudinal patterns in prevalence were found. Nausea: prevalence fell sharply from 42.5% at T1 to 5.9% at T3 (a reduction of 36.6 percentage points), representing the largest decrease observed across the symptom set. Vomiting: similarly reduced from 27.5% to 5.7% by 12 months. Fatigue: showed a more complex trajectory, improving to 45.7% at T2 before returning to 77.1% at T3, the highest prevalence observed at that time point. Dry skin: prevalence rose from 35.0% at T1 to 45.7% at T3, representing a notable increase over the follow-up period. Overall perceived health remained a consistently high-burden item at all time points (80.0%, 78.3%, 61.8%), with a modest improvement by T3. Symptom-level prevalence at each time point is presented and displayed graphically in Figure 3 and Table 4.

Supplementary figures display the change in prevalence per symptom between T1 and T3 (Figure S2) and the distribution of symptom severity scores at each time point for selected high-burden symptoms (Figure S3).

### 3.1. Subgroup Analysis

Two-way analysis of variance was used to examine the effects of sex and diabetes mellitus (DM) status on mean symptom severity scores at baseline. A significant effect of DM status was identified for dyspnea (*p* < 0.001), indicating that patients with DM reported greater breathlessness compared with those without DM. No statistically significant interaction between sex and DM status was found for any symptom after adjustment for multiple comparisons. Full results are presented in Supplementary Table S1.

Two-way ANOVA examining the effects of age group and vascular access type (fistula vs. catheter) identified significant differences in symptom severity for three symptoms: pain (vascular access effect: *p* = 0.017), fatigue (*p* = 0.002), and nausea (*p* = 0.008). In each case, patients receiving HD via central venous catheter reported higher symptom severity than those with an arteriovenous fistula. Full results are presented in Supplementary Table S2.

### 3.2. Correlations with Sociodemographic and Clinical Variables

No statistically significant associations between per-patient mean symptom severity and any of the sociodemographic or clinical variables examined (dialysis vintage, household density, treatment frequency, religiosity, education level, occupational status, financial satisfaction, housing satisfaction, and religion) were identified after Benjamini–Hochberg false discovery rate correction (all FDR-adjusted *p* > 0.05). Complete Spearman ρ and Kendall τb correlation matrices are provided in Supplementary Tables S3 and S4, and the pattern of associations is visualized in Supplementary Figure S5.

To characterize symptom change over 12 months, linear mixed-effects models were fitted for each symptom cluster, with mean cluster severity as the outcome, time (in years from baseline) as the fixed predictor, and a random intercept per patient to account for repeated measures. Five clusters were modelled: Emotional-Physical, Fatigue-Sleep, Gastrointestinal, Neurological/Lower-limb, and Sensory-Skin.

**Figure 4:**
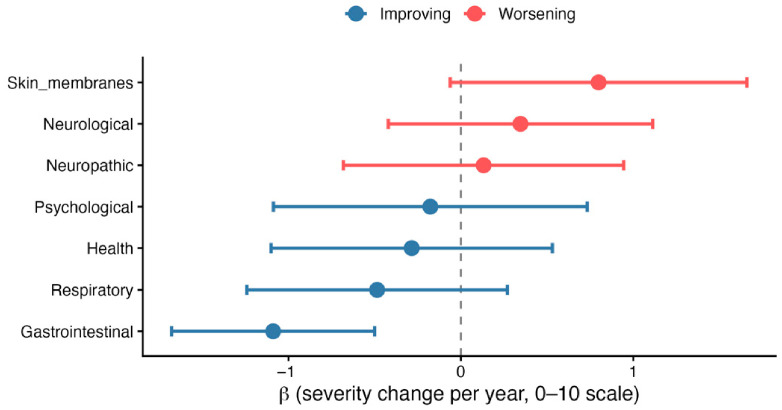
Domain-level symptom trajectories over time. Forest plot of linear mixed-effects estimates (β ± 95% CI) for annual change in symptom severity (0–10 scale) across analytic symptom domains. Negative values indicate a decrease in symptom severity over time. These domains represent component symptom groupings that contribute to the five higher-order clinical symptom clusters analyzed longitudinally and presented in Table 3.

Model results are presented in Figure 4. In the sensitivity analysis adjusting for DM status, the direction and magnitude of all cluster trajectories were unchanged (Supplementary Table S5). The Gastrointestinal cluster remained the only cluster with a statistically significant improvement over time (β = −1.031, 95% CI −1.639 to −0.423, *p* < 0.001), nearly identical to the unadjusted estimate (β = −1.057). DM status was itself significantly associated with Gastrointestinal cluster scores (β = 0.712, *p* = 0.032), indicating that patients with diabetes reported higher gastrointestinal symptom burden at baseline.

**Figure 5:**
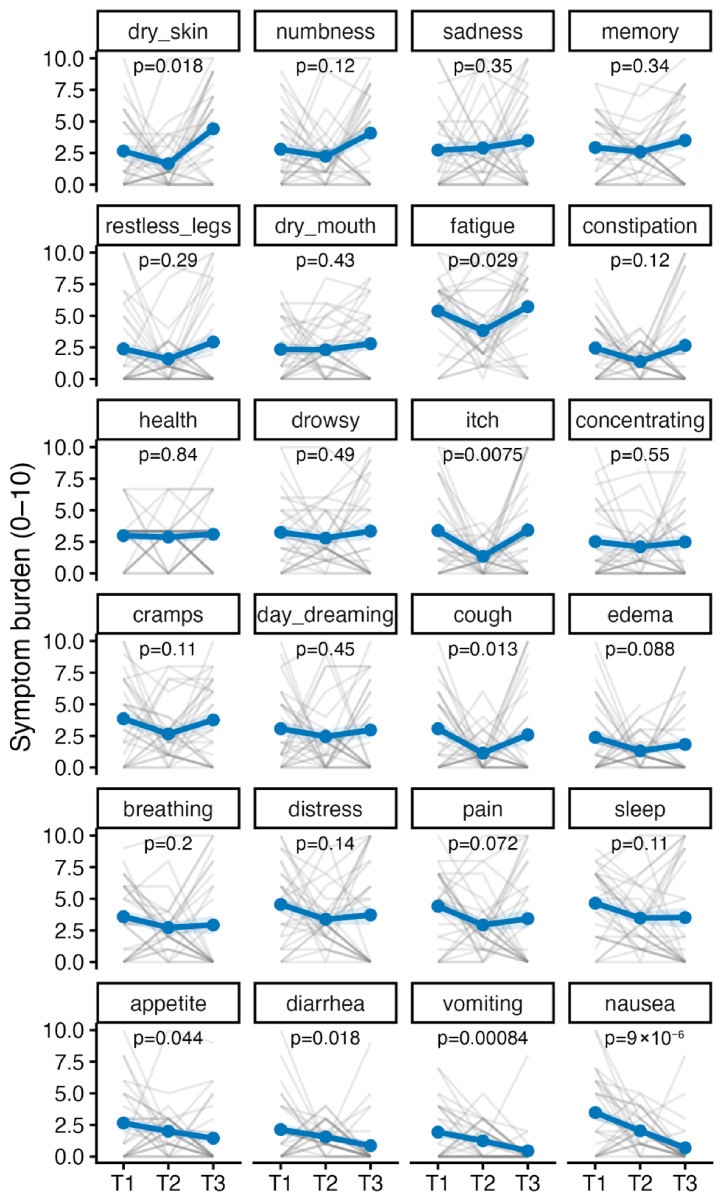
Symptom burden over time, ordered by trajectory. Grey lines = individual patients; blue = mean ± SE.

The Gastrointestinal cluster was the only cluster to show a statistically significant trajectory over time in the unadjusted model (β = −0.914, *p* = 0.003), indicating a mean decrease of approximately 0.91 points per year on the 0–10 scale. No significant change over time was observed for the remaining clusters: Emotional-Physical (β = 0.114, *p* = 0.790), Fatigue-Sleep (β = 0.075, *p* = 0.865), Neurological/Lower-limb (β = −0.361, *p* = 0.317), and Sensory-Skin (β = 0.267, *p* = 0.427). Individual-level trajectories are illustrated in Figure 5, which highlights considerable within-cluster heterogeneity in symptom change across patients.

## 4. Discussion

This prospective, longitudinal, observational, nurse-led study addresses existing gaps by broadening our understanding of cluster evolution in HD patients over time, as well as the limited integration of individual patient characteristics into cluster analyses. The theoretical contribution of this study is in identifying and organizing symptoms by distinct clusters in HD.

This study demonstrates that, regardless of socio-demographics, patients receiving HD maintenance experience a persistently high and fluctuating symptom burden. The symptoms reported by the sample patients align with previously acknowledged symptoms in CKD for HD patients, changing in severity and decreasing QoL, including depression, anxiety, and complications such as repetitive infections, pain, anemia, and fatigue [20,21,22].

At baseline, the most prevalent symptom was fatigue, followed by sleep disturbance and worry or distress. Also, HD patients with DM reported greater breathlessness at baseline compared to HD patients without DM. Fatigue, impaired overall perceived health, sleep disturbance, and psychological distress emerged as the most severe and consistently reported symptoms across time points. Although a transient reduction in symptom severity was observed at six months, this improvement was not sustained at 12 months, underscoring the dynamic nature of symptom experience in this population. The pattern of clinically significant symptoms mirrored this trajectory, with an initial decline followed by a return toward baseline levels, suggesting that symptom relief may be temporary or sensitive to changes in clinical status, treatment conditions, or life circumstances.

This finding suggests that symptom co-occurrence patterns are dynamic in the HD population and that cluster structures derived at a single time point may not reliably reflect symptom burden at later assessments. These findings contrast those of a previous study [6], where patient symptoms across clusters were stable rather than co-occurring and dynamic. In our study, only symptoms in the uremic cluster were stable (e.g., nausea, vomiting, weight loss, difficulty concentrating, fatigue). The temporal instability observed in our study between time points indicates that symptom interrelationships are not fixed. Instead, patients’ symptom profiles reorganize substantially over time, likely reflecting evolving comorbidities, treatment adaptations, and psychosocial factors. Seasonality may also have contributed to the observed temporal changes, as assessments occurred in different seasonal windows. Because the study was not designed to disentangle seasonal effects from adaptation-related changes, this interpretation is hypothesis-generating. This instability highlights the limitations of relying on static symptom groupings for clinical decision-making and supports the need for repeated assessment rather than one-time profiling.

The hierarchical clustering analysis identified five clinically interpretable symptom groupings, reflecting multidimensional domains of systemic, fatigue-related, gastrointestinal, sensory–neurological, and dermatological/attentional symptoms. The Gastrointestinal cluster was the only cluster showing a statistically significant trajectory over time, while the other clusters showed no change over time, supporting another study [6]. Further, the cluster structures derived at a single time point may not reliably reflect symptom burden at later assessments. The burden of the perceived overall health, however, remained a high-burden symptom across time points. The Individual trajectories of patients also highlighted considerable within-cluster heterogeneity in symptom change across patients.

Longitudinal modelling further demonstrated that only the gastrointestinal cluster showed a significant improvement over the 12 months, driven by marked reductions in nausea and vomiting. This finding may reflect improved dialysis adequacy, medication adjustments, or dietary stabilization over time. In contrast, the absence of significant change in fatigue, sleep, emotional, physical pain, and sensory skin clusters suggests that these domains are more refractory to routine clinical management and may require targeted interventions. The substantial heterogeneity in individual trajectories reinforces the importance of personalized symptom monitoring rather than relying solely on group-level trends.

Subgroup analyses revealed that DM was associated with greater dyspnea, consistent with the cardiopulmonary complications commonly observed in diabetic dialysis patients [23,24]. Additionally, patients dialyzing via central venous catheter reported higher severity of pain, fatigue, and nausea compared with those using arteriovenous fistulas, aligning with evidence that catheter use is associated with greater inflammation, infection risk, and overall morbidity [25]. These findings emphasize the need to consider patient characteristics and vascular access type when interpreting symptom reports and planning supportive care.

The findings highlight the potential role of nephrology nurse specialists within value-based healthcare frameworks. Through ongoing care of high-risk patients, longitudinal symptom monitoring, early recognition of symptom escalation, and structured vascular access assessment, nurses may contribute to improved care coordination and more tailored, cluster-informed supportive care. Such activities may have implications for patient experience and healthcare utilization and warrant further evaluation in future studies [10,11,12].

Although a formal cost-effectiveness analysis was beyond the scope of this study, these VBHC-relevant activities can be evaluated in future work by pairing measurable resource use (e.g., nurse time and unplanned healthcare utilization) with patient-centered outcomes (e.g., symptom burden and QoL) to quantify value.

Nurses can also recognize subtle signs of depression and allow for early psychological interventions to keep the patient adherent to their life-saving treatments. As the first line of defense against the complications of CKD, which increase costs and reduce QoL, nurses should be central in feedback loops to continuously improve learning and processes of care [26].

## 5. Conclusions

The study results underscore the complexity and variability of symptom experiences in HD patients. The observed temporal instability of symptom clusters and the heterogeneity of individual trajectories emphasize the importance of routine, longitudinal symptom assessment and flexible, patient-centered management strategies by nephrology nurse specialists, which may inform the future development of value-based, nurse-led care approaches.

## 6. Study Limitations

The novel findings of this study are not without limitations. This study was conducted in a single medical center, limiting generalizability. Additionally, symptom structures may change across countries, dialysis modalities, and healthcare systems.

In addition, the rare cohort represents a convenience sample recruited based on feasibility within a single clinical setting, which may further limit generalizability.

The sample size in this study is modest relative to the analytical complexity of the multivariate (clustering and PCA) and longitudinal (mixed-effects) components. Given real-world recruitment and retention constraints in a chronic HD population, we interpret the clustering and PCA findings as exploratory and hypothesis-generating, intended to summarize multivariate structure and support descriptive interpretation rather than to provide definitive subgroup classification. Multivariate post hoc pattern-discovery approaches (e.g., PCA and clustering) can be sensitive to overparameterization and sample-specific instability when the number of participants is limited.

Therefore, we emphasize effect sizes and uncertainty, and highlight the need for replication in larger, preferably multi-center cohorts to confirm the stability and generalizability of the observed temporal patterns. Also, because attrition was largely health-related (clinical deterioration, transplantation, or death), later time point results may disproportionately reflect clinically stable survivors and should be interpreted with caution. 

## 7. Direction for Future Studies

Future research should explore mechanisms underlying symptom reorganization over time and evaluate interventions tailored to persistent high burden domains such as fatigue, emotional distress, and sleep disturbance. The marked reorganization of symptom clusters over 12 months suggests that symptoms evolve in response to changing physiological, psychological, and treatment-related factors. Therefore, future studies are called to track biological markers (inflammation, dialysis adequacy, nutritional status) alongside symptoms to identify drivers of cluster instability. In parallel, future studies should prospectively capture utilization, which is central to VBHC in nursing (e.g., nurse time, additional contacts/referrals, missed sessions, and unplanned healthcare utilization such as ED visits and hospitalizations). Pairing these utilization/cost drivers with patient-centered outcomes will enable formal cost-effectiveness and budget impact analyses of cluster-informed nurse-led care. Future studies may also consider alternative study designs, such as larger multi-center cohorts, longer follow-up periods, or interventional approaches, to further examine symptom cluster dynamics and their clinical implications.

Also, more frequent symptom assessments (e.g., weekly digital reporting) should be used to capture short-term fluctuations and transitions between symptom states. Further, considering the substantial heterogeneity in patient-level trajectories, future studies may apply machine learning models to identify subgroups with distinct symptom evolution patterns. This will facilitate early warning models that predict worsening fatigue, emotional distress, or sleep disturbance. As for interventions, future studies can test multimodal interventions (exercise, cognitive behavioral therapy, sleep hygiene programs, intradialytic activity) tailored to these domains, and evaluate whether cluster-based interventions, rather than symptom-specific interventions, produce broader improvements.

## Figures and Tables

**Table 1:** Demographic and clinical characteristics of the study rare cohort (n = 69).

Characteristic	Value
Age, mean ± SD (range), years	63.5 ± 15.7 (24–87)
Sex, n (%)	
Male	48 (69.6)
Female	21 (30.4)
Diabetes mellitus, n (%)	34 (47.9)
Vascular access, n (%)	
Arteriovenous fistula (%)	50 (72.4)
Central venous catheter (%)	19 (27.5)
Prior kidney transplantation, n (%)	11 (15.5)
Dialysis vintage, mean ± SD (range), years	5.66 ± 4.73 (2–25)

**Table 2:** Mean (SD) symptom severity scores (0–10 scale) at each time point. Values are mean ± SD on a 0–10 numeric rating scale. at T1, T2, and T3, sorted by T1 mean severity. Symptoms sorted by T1 mean severity (highest first). Overall perceived health rescaled from 1–4 to 0–10.

Symptom	T1, Baseline (n = 45)	T2, ~6 Months (n = 42)	T3, ~12 Months (n = 31)
Fatigue	5.13 ± 3.08	3.92 ± 2.56	5.33 ± 3.37
Overall perceived health	4.83 ± 1.35	4.53 ± 1.48	4.28 ± 1.89
Worry/distress	4.18 ± 3.22	3.06 ± 2.23	3.39 ± 4.09
Sleep disturbance	4.11 ± 3.11	3.30 ± 2.41	3.80 ± 3.90
Pain	3.96 ± 2.93	2.89 ± 2.40	2.71 ± 3.75
Muscle cramps	3.53 ± 3.17	2.74 ± 2.39	3.22 ± 3.35
Dyspnea	3.51 ± 3.00	2.68 ± 2.16	2.50 ± 3.71
Pruritus	3.33 ± 3.26	2.08 ± 2.25	3.50 ± 3.93
Drowsiness	3.11 ± 3.02	2.60 ± 2.24	3.26 ± 3.50
Memory impairment	3.02 ± 2.67	2.83 ± 2.00	3.11 ± 3.43
Nausea	2.91 ± 3.07	2.15 ± 1.76	0.67 ± 1.62
Dry mouth	2.73 ± 2.82	2.26 ± 2.23	3.04 ± 3.17
Cough	2.67 ± 2.90	1.75 ± 1.91	2.02 ± 3.44
Decreased appetite	2.64 ± 2.75	2.42 ± 2.32	1.72 ± 2.93
Dry skin	2.62 ± 2.72	2.28 ± 2.25	4.13 ± 3.98
Numbness/tingling	2.56 ± 2.82	1.96 ± 2.10	3.15 ± 3.53
Inattention	2.53 ± 2.94	2.15 ± 2.44	2.39 ± 3.33
Sadness	2.49 ± 2.89	2.47 ± 2.63	2.89 ± 3.71
Constipation	2.38 ± 2.50	1.94 ± 1.59	2.70 ± 3.50
Poor concentration	2.38 ± 2.81	2.25 ± 2.25	2.63 ± 3.28
Restless legs	2.33 ± 2.96	1.91 ± 1.96	2.24 ± 3.60
Diarrhea	2.22 ± 2.72	1.98 ± 1.70	0.93 ± 1.88
Leg edema	2.07 ± 2.69	1.62 ± 1.46	1.69 ± 2.59
Vomiting	1.64 ± 2.21	1.53 ± 1.58	0.83 ± 2.20

**Table 3:** Symptom clusters derived from hierarchical clustering.

Cluster	Constituent Symptoms	Clinical Theme
1	Pain, Worry/distress, Sadness, Numbness/tingling, Decreased appetite, Dyspnea, Memory impairment	Emotional-Physical
2	Overall perceived health, Drowsiness, Dry mouth, Muscle cramps, Fatigue, Sleep disturbance	Fatigue-Sleep
3	Constipation, Diarrhea, Nausea, Vomiting	Gastrointestinal
4	Restless legs, Leg edema, Daydreaming/inattention	Neurological/Lower-limb
5	Dry skin, Pruritus, Cough, Poor concentration	Sensory-Skin

**Table 4:** Prevalence of Clinically Significant Symptoms. Clinically significant symptom burden was defined as a severity rating ≥ 4 on the 0–10 scale, consistent with thresholds used in prior dialysis symptom research.

Symptom	T1 Baseline	T2 (~6 Months)	T3 (~12 Months)
Overall perceived health	80%	78.3%	61.8%
Fatigue	75%	45.7%	77.1%
Sleep disturbance	60%	32.6%	40%
Worry/distress	60%	34.8%	45.7%
Pain	55%	26.1%	44.1%
Dyspnea	52.5%	26.1%	34.3%
Memory impairment	45%	30.4%	47.1%
Nausea	42.5%	21.7%	5.9%
Muscle cramps	42.5%	26.1%	42.9%
Cough	40%	15.2%	28.6%
Decreased appetite	37.5%	23.9%	20%
Drowsiness	37.5%	32.6%	45.7%
Dry mouth	35%	21.7%	40%
Numbness/tingling	35%	15.2%	48.6%
Constipation	35%	13%	31.4%
Inattention	35%	15.2%	34.3%
Dry skin	35%	23.9%	45.7%
Pruritus	35%	17.4%	31.4%
Sadness	32.5%	21.7%	40%
Restless legs	32.5%	13%	34.3%
Poor concentration	32.5%	21.7%	34.3%
Vomiting	27.5%	8.7%	5.7%
Diarrhea	27.5%	15.2%	11.4%
Leg edema	25%	13%	23.5%

## Data Availability

The raw data of this article are not available for external use.

## Supplementary Materials

The following supporting information can be downloaded at https://www.mdpi.com/article/10.3390/healthcare14101375/s1. Supplementary Figure S1. Cluster Elbow Plot. Supplementary Figure S2. Prevalence Change from T1 to T3. Supplementary Figure S3. Cluster Stability Heat maps (T1, T2, T3). Supplementary Figure S4. (a–d) GDM2 Cluster Stability Analysis. Supplementary Figure S5. Sociodemographic Correlation Heat map. Supplementary Figure S6. Silhouette analysis of the cluster solution. Supplementary Table S1: Subgroup Analysis by Sex and Diabetes Status. Supplementary Table S2: Subgroup Analysis by Age Group and Vascular Access Type. Supplementary Table S3. Spearman ρ correlation coefficients between per-patient mean symptom severity and continuous sociodemographic variables. Supplementary Table S4. Kendall τb correlation coefficients between per-patient mean symptom severity and ordinal sociodemographic variables. Supplementary Table S5. Sensitivity analysis with DM-adjusted linear mixed-effects models for cluster symptom trajectories.

## Abbreviations

The following abbreviations are used in this manuscript:

HD	HD
CKD	chronic kidney disease
QoL	Quality of Life
DM	Diabetes Mellitus
VBHC	Value-Based Health Care
PCA	Principal Component Analysis
MDASI	MD Anderson Symptom Inventory
ARI	Adjusted Rand Index
