# Supplementary material for: Symptom Clusters and Longitudinal Progression in Chronic Hemodialysis Patients: A Prospective Single-Center Study
Source: Healthcare (Basel). 2026 May 18;14(10):1375. doi: 10.3390/healthcare14101375 (PMC13205381; doi:10.3390/healthcare14101375)
Supplement: Supplementary file 1 [file healthcare-14-01375-s001.zip › Supplementary Figure Legends.pdf]

## Supplementary Figure Legends

**Supplementary Figure S1. Cluster Elbow Plot.** Within-cluster sum of squares (WSS) for k = 1–15 clusters computed at baseline (T1), used to select k = 5 as the optimal number of symptom clusters. The elbow at k = 5 (highlighted in red) indicates the point of diminishing returns in within-cluster variance reduction.

**Supplementary Figure S2. Prevalence Change from T1 to T3.** Change in the prevalence of clinically significant symptoms (severity ≥4/10) between baseline (T1) and 12-month follow-up (T3), expressed as percentage-point differences. Positive values indicate increased prevalence; negative values indicate decreased prevalence. Symptoms are ordered by magnitude of change.

**Supplementary Figure S3. Cluster Stability Heatmaps (T1, T2, T3).** Annotated patient × symptom heatmaps displaying the five-cluster solution applied independently at each time point. Panels A (Baseline/T1), B (6 months/T2), and C (12 months/T3) show hierarchical clustering solutions (Ward D2 linkage, Canberra distance) with patient cluster membership indicated by color-coded annotation bars. Moderate cluster agreement was observed between T1 and T2 (ARI = 0.588), while agreement was absent between T1 and T3 (ARI = 0.023) and between T2 and T3 (ARI = −0.013).

**Supplementary Figure S4. GDM2 Cluster Stability Analysis.** Stability of the five-cluster solution assessed using the Generalized Dissimilarity Metric 2 (GDM2; clusterSim package). A symptom × patient matrix was submitted to GDM2 to compute a pairwise symptom dissimilarity matrix, which was then projected to two dimensions by classical multidimensional scaling. Panels a–d show results for T1 (Baseline), T2 (~6 months), T3 (~12 months), and per-patient mean scores across all time points, respectively. Symptoms are color-coded by cluster assignment.

**Supplementary Figure S5. Sociodemographic Correlation Heatmap.** Heatmap of Spearman $\rho$ and Kendall $\tau^b$ correlations between per-patient mean symptom severity and sociodemographic and clinical variables (dialysis vintage, household density, treatment frequency, religiosity, education, occupational status, financial satisfaction, housing satisfaction). Color intensity represents correlation strength; all associations were non-significant after Benjamini–Hochberg false discovery rate correction (all FDR-adjusted $p > 0.05$).

**Supplementary Figure S6. Silhouette analysis of the cluster solution.** The average silhouette width (ASW) is shown across different numbers of clusters, with a maximal value at k = 5 (ASW = 0.08), indicating weak separation between clusters.
