# Supplementary material for: Symptom Clusters and Longitudinal Progression in Chronic Hemodialysis Patients: A Prospective Single-Center Study
Source: Healthcare (Basel). 2026 May 18;14(10):1375. doi: 10.3390/healthcare14101375 (PMC13205381; doi:10.3390/healthcare14101375)
Supplement: Supplementary file 1 [file healthcare-14-01375-s001.zip › Supplementary Figure S2 - Prevalence Change T1 to T3.pdf]

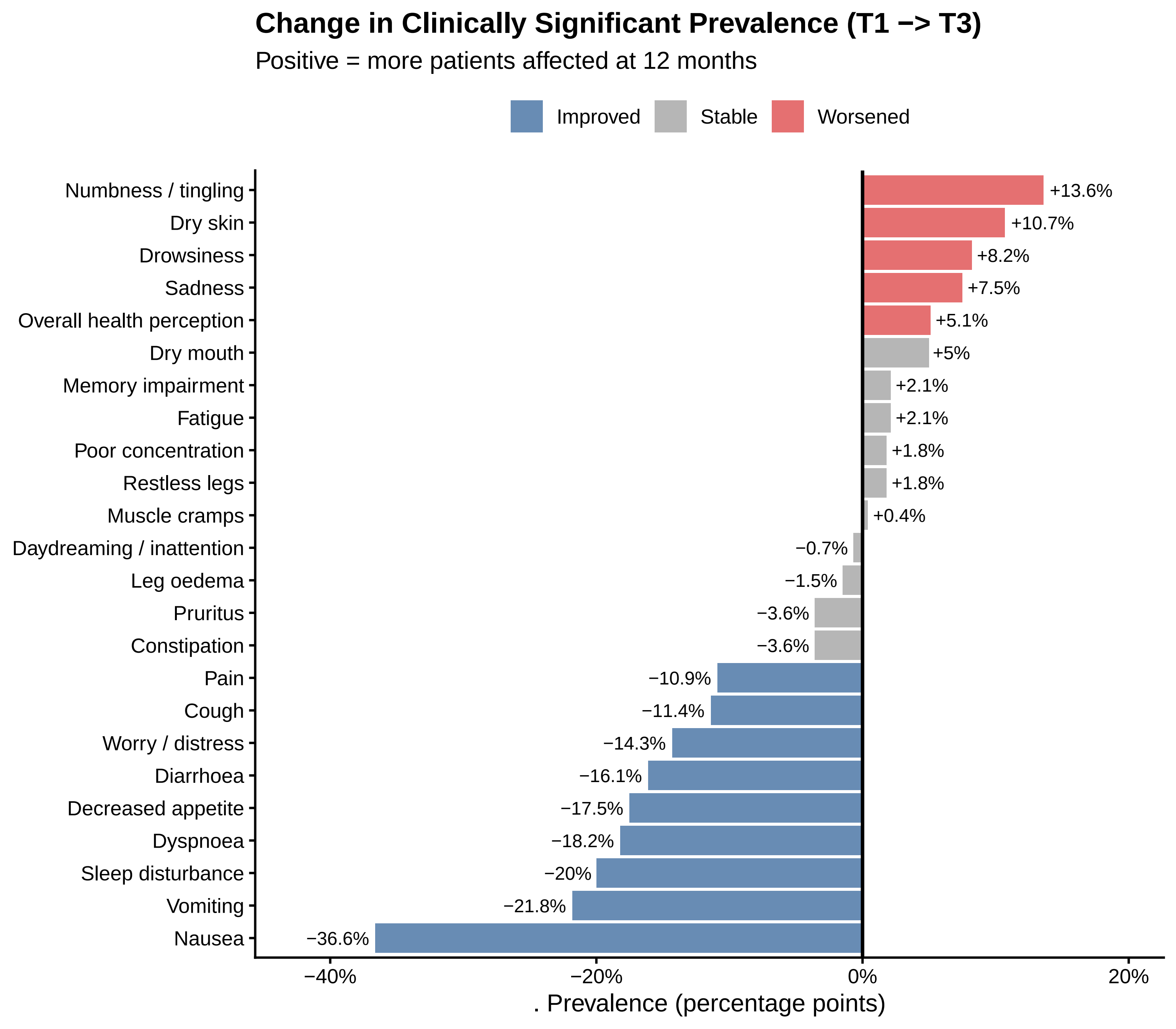
Change in Clinically Significant Prevalence (T1 −> T3)
Positive = more patients affected at 12 months
Improved
Stable
Worsened
Numbness / tingling
Dry skin
Drowsiness
Sadness
Overall health perception
Dry mouth
Memory impairment
Fatigue
Poor concentration
Restless legs
Muscle cramps
Daydreaming / inattention
Leg oedema
Pruritus
Constipation
Pain
Cough
Worry / distress
Diarrhoea
Decreased appetite
Dyspnoea
Sleep disturbance
Vomiting
Nausea
+13.6%
+10.7%
+8.2%
+7.5%
+5.1%
+5%
+2.1%
+2.1%
+1.8%
+1.8%
+0.4%
−0.7%
−1.5%
−3.6%
−3.6%
−10.9%
−11.4%
−14.3%
−16.1%
−17.5%
−18.2%
−20%
−21.8%
−36.6%
−40%
−20%
0%
20%
. Prevalence (percentage points)
