# Supplementary material for: Symptom Clusters and Longitudinal Progression in Chronic Hemodialysis Patients: A Prospective Single-Center Study
Source: Healthcare (Basel). 2026 May 18;14(10):1375. doi: 10.3390/healthcare14101375 (PMC13205381; doi:10.3390/healthcare14101375)
Supplement: Supplementary file 1 [file healthcare-14-01375-s001.zip › Supplementary Figure S5 - Sociodemographic Correlation Heatmap (1).pdf]

Symptom severity (mean T1−T3) vs. sociodemographic variables

Spearman .: dialysis vintage, household density | Kendall .b: all ordinal variables | * BH−adj p < 0.05; . BH−adj p < 0.10

	Dialysis vintage (months)	Treatment frequency (per week)	Religiosity	Education level	Household density (persons/room)	Occupational status	Financial satisfaction	Housing satisfaction
Pain	0.17	0.18	0.01	0.02	−0.01	0.05	−0.06	−0.08
Fatigue	−0.06	0.17	0.09	−0.08	0.10	0.05	0.07	0.07
Nausea	−0.08	0.14	−0.05	−0.01	−0.02	0.03	−0.03	−0.14
Sleep disturbance	−0.01	0.16	0.14	−0.03	−0.14	0.17	−0.01	−0.01
Worry / distress	−0.13	0.18	0.03	−0.11	−0.05	−0.10	−0.11	−0.14
Dyspnea	−0.09	0.22	0.10	−0.08	−0.23	0.05	0.18	0.15
Memory impairment	0.04	0.09	−0.07	0.04	0.14	0.18	0.12	0.03
Decreased appetite	0.15	0.14	−0.02	−0.12	0.06	0.06	−0.01	−0.06
Drowsiness	−0.01	0.13	0.04	−0.02	−0.14	0.09	0.22	0.12
Dry mouth	0.07	0.11	0.10	−0.10	−0.03	0.11	0.15	0.02
Sadness	−0.02	0.22	−0.03	−0.10	−0.10	0.15	0.03	−0.06
Vomiting	0.01	0.22	−0.01	−0.05	−0.22	−0.02	−0.04	−0.08
Numbness / tingling	−0.07	0.18	0.12	−0.06	−0.17	0.09	0.04	−0.07
Constipation	0.01	0.07	0.21	−0.06	−0.20	0.11	0.10	0.02
Diarrhea	−0.05	0.16	−0.02	−0.02	−0.26	−0.01	0.14	0.19
Muscle cramps	−0.03	0.05	0.12	−0.07	0.02	0.13	0.05	0.01
Leg edema	0.07	−0.04	0.06	−0.02	−0.04	−0.03	0.04	−0.02
Daydreaming / inattention	−0.01	0.06	−0.02	−0.06	0.07	0.04	0.08	−0.01
Restless legs	0.14	0.17	0.07	0.00	−0.26	0.15	0.14	0.10
Cough	0.16	0.18	0.14	−0.08	−0.29	0.10	0.27	0.09
Poor concentration	0.21	0.02	−0.08	−0.06	0.11	0.01	0.14	−0.01
Dry skin	0.09	0.06	0.15	0.02	−0.41.	0.03	0.26	0.29
Pruritus	0.01	−0.04	0.15	−0.01	−0.21	0.02	0.17	0.20
Overall health perception	0.07	0.00	0.01	0.18	−0.16	−0.07	−0.00	0.14

. / .b: 1.0, 0.5, 0.0, −0.5, −1.0
