# Supplementary material for: Symptom Clusters and Longitudinal Progression in Chronic Hemodialysis Patients: A Prospective Single-Center Study
Source: Healthcare (Basel). 2026 May 18;14(10):1375. doi: 10.3390/healthcare14101375 (PMC13205381; doi:10.3390/healthcare14101375)
Supplement: Supplementary file 1 [file healthcare-14-01375-s001.zip › Supplementary Table S1 - Subgroup Analysis by Sex and Diabetes Status (1).pdf]

Supplementary Table S1: Subgroup Analysis by Sex and Diabetes Status

Mean (SD) symptom severity by sex × diabetes status subgroups with two-way ANOVA p-values.

Table 4. Mean (SD) symptom severity by sex × diabetes status subgroups with two-way ANOVA p-values (significant p-values highlighted)

Symptom	Male, DM	Male, No DM	Female, DM	Female, No DM	p (sex)	p (DM)
Pain	3.93 ± 1.92	2.88 ± 2.52	2.24 ± 2.07	1.73 ± 2.34	0.026	0.114
Fatigue	5.15 ± 1.94	4.44 ± 2.67	4.60 ± 2.65	3.83 ± 2.77	0.445	0.229
Nausea	2.44 ± 1.95	1.69 ± 1.56	1.86 ± 1.80	0.97 ± 1.26	0.204	0.055
Sleep disturbance	4.01 ± 2.46	3.85 ± 2.98	3.47 ± 2.56	3.30 ± 2.40	0.452	0.799
Worry / distress	3.86 ± 2.27	2.64 ± 2.86	3.42 ± 3.08	3.87 ± 3.12	0.524	0.319
Dyspnea	4.18 ± 2.37	1.96 ± 2.51	2.90 ± 1.93	1.30 ± 1.29	0.209	<0.001
Memory impairment	3.92 ± 2.24	2.48 ± 2.53	2.88 ± 2.19	2.27 ± 1.69	0.408	0.038
Decreased appetite	2.92 ± 2.28	2.31 ± 2.46	2.01 ± 2.15	1.10 ± 1.47	0.096	0.193
Drowsiness	3.53 ± 2.55	2.22 ± 2.30	2.36 ± 1.72	3.67 ± 2.96	0.784	0.435
Dry mouth	3.04 ± 2.41	2.70 ± 2.53	2.86 ± 2.84	3.13 ± 2.65	0.836	0.820
Sadness	2.82 ± 2.07	1.79 ± 1.96	2.76 ± 2.79	2.57 ± 2.86	0.451	0.175
Vomiting	2.12 ± 1.77	1.22 ± 2.23	1.12 ± 1.43	0.67 ± 0.90	0.150	0.093
Numbness / tingling	2.88 ± 1.75	1.95 ± 2.27	2.17 ± 1.85	2.03 ± 1.78	0.644	0.170
Constipation	3.09 ± 1.96	2.01 ± 2.31	2.79 ± 2.59	1.60 ± 1.19	0.688	0.034
Diarrhea	2.67 ± 1.93	1.51 ± 1.80	1.25 ± 1.32	1.10 ± 1.11	0.066	0.046
Muscle cramps	3.39 ± 2.58	3.19 ± 2.71	2.32 ± 1.72	2.80 ± 2.03	0.243	0.975
Leg edema	1.94 ± 1.37	1.73 ± 2.07	1.60 ± 1.24	1.63 ± 2.06	0.646	0.763
Daydreaming / inattention	2.02 ± 1.24	2.04 ± 2.49	2.18 ± 2.22	2.73 ± 2.64	0.477	0.724
Restless legs	2.63 ± 1.81	1.48 ± 1.86	2.19 ± 1.60	1.53 ± 2.70	0.882	0.037
Cough	3.01 ± 2.31	1.72 ± 1.88	1.29 ± 2.11	1.37 ± 1.33	0.074	0.085
Poor concentration	2.51 ± 2.37	2.49 ± 1.96	1.97 ± 1.90	2.70 ± 2.90	0.732	0.678
Dry skin	3.77 ± 2.68	2.02 ± 2.28	3.78 ± 3.17	3.17 ± 2.74	0.280	0.034
Pruritus	3.33 ± 2.76	2.59 ± 2.67	3.35 ± 2.84	3.93 ± 3.62	0.345	0.654
Overall perceived health	4.62 ± 1.33	4.49 ± 1.12	4.24 ± 1.27	4.08 ± 1.21	0.228	0.636

Values are mean ± SD of scores averaged across all three time points. p-values from two-way ANOVA (sex + diabetes status as independent factors). Red = $p < 0.05$.
