# Supplementary material for: Symptom Clusters and Longitudinal Progression in Chronic Hemodialysis Patients: A Prospective Single-Center Study
Source: Healthcare (Basel). 2026 May 18;14(10):1375. doi: 10.3390/healthcare14101375 (PMC13205381; doi:10.3390/healthcare14101375)
Supplement: Supplementary file 1 [file healthcare-14-01375-s001.zip › Supplementary Table S2 - Subgroup Analysis by Age Group and Vascular Access.pdf]

Supplementary Table S2: Subgroup Analysis by Age Group and Vascular Access Type
Mean (SD) symptom severity by age group × vascular access subgroups with two-way ANOVA p-values.

Table 5. Mean (SD) symptom severity by age group × vascular access subgroups with two-way ANOVA p-values (significant p-values highlighted)

**Symptom**	**Younger, Shunt**	**Younger, Catheter**	**Older, Shunt**	**Older, Catheter**	**p (age)**	**p (access)**
Pain	4.13 ± 2.53	1.11 ± 1.47	2.64 ± 1.89	3.21 ± 2.67	0.576	0.017
Fatigue	4.93 ± 2.54	3.53 ± 2.97	5.33 ± 1.90	2.31 ± 1.75	0.603	0.002
Nausea	2.80 ± 2.03	1.04 ± 1.42	1.58 ± 1.38	1.14 ± 1.18	0.084	0.008
Sleep disturbance	3.92 ± 2.65	2.18 ± 2.11	4.17 ± 2.56	4.19 ± 3.24	0.171	0.163
Worry / distress	4.41 ± 2.78	2.49 ± 3.36	2.96 ± 2.35	2.62 ± 2.77	0.208	0.095
Dyspnea	2.77 ± 2.00	1.86 ± 3.08	3.27 ± 2.55	1.38 ± 1.42	0.444	0.049
Memory impairment	3.23 ± 2.42	2.40 ± 2.74	2.85 ± 2.23	3.24 ± 1.97	0.989	0.625
Decreased appetite	3.06 ± 2.71	1.82 ± 2.35	2.12 ± 1.94	1.05 ± 1.04	0.185	0.057
Drowsiness	3.98 ± 2.63	2.11 ± 2.59	2.56 ± 2.10	1.60 ± 1.66	0.094	0.022
Dry mouth	4.02 ± 2.73	2.31 ± 2.94	2.53 ± 1.98	1.79 ± 2.19	0.081	0.053
Sadness	3.10 ± 2.52	1.22 ± 1.79	2.20 ± 2.04	2.74 ± 2.76	0.812	0.173
Vomiting	2.20 ± 2.07	1.61 ± 2.91	0.85 ± 0.88	0.60 ± 0.83	0.007	0.363
Numbness / tingling	2.90 ± 2.22	0.97 ± 1.35	2.41 ± 1.86	1.93 ± 1.92	0.841	0.015
Constipation	2.55 ± 2.21	1.33 ± 1.72	2.38 ± 1.79	3.88 ± 3.38	0.292	0.903
Diarrhea	2.09 ± 1.87	1.75 ± 1.66	1.76 ± 1.84	0.55 ± 0.63	0.281	0.144
Muscle cramps	3.22 ± 2.00	3.22 ± 3.33	3.11 ± 2.47	1.88 ± 1.69	0.549	0.443
Leg edema	1.65 ± 1.64	2.12 ± 2.68	1.68 ± 1.41	1.74 ± 1.45	0.771	0.536
Daydreaming / inattention	2.66 ± 2.41	2.24 ± 2.63	1.84 ± 1.70	1.74 ± 2.10	0.185	0.624
Restless legs	2.41 ± 2.45	0.90 ± 1.40	1.88 ± 1.52	2.55 ± 2.31	0.782	0.277
Cough	2.08 ± 2.02	1.32 ± 1.85	2.29 ± 2.34	1.40 ± 1.22	0.547	0.153
Poor concentration	3.19 ± 2.48	2.49 ± 2.65	2.11 ± 1.74	1.31 ± 1.44	0.057	0.208
Dry skin	2.89 ± 2.70	1.78 ± 2.56	3.59 ± 2.49	3.07 ± 3.51	0.127	0.241
Pruritus	2.54 ± 2.18	2.72 ± 3.01	3.67 ± 3.11	3.55 ± 3.48	0.132	0.944
Overall health perception	4.39 ± 1.32	3.89 ± 1.25	4.60 ± 1.13	4.76 ± 1.04	0.158	0.498

Values are mean ± SD of scores averaged across all three time points. Age groups defined by median split. p-values from two-way ANOVA (age group + vascular access as independent factors). Red = $p < 0.05$.
