# Supplementary material for: Symptom Clusters and Longitudinal Progression in Chronic Hemodialysis Patients: A Prospective Single-Center Study
Source: Healthcare (Basel). 2026 May 18;14(10):1375. doi: 10.3390/healthcare14101375 (PMC13205381; doi:10.3390/healthcare14101375)
Supplement: Supplementary file 1 [file healthcare-14-01375-s001.zip › Supplementary Table S3 - Spearman Correlation Matrix.pdf]

Supplementary Table S3. Spearman ρ correlation coefficients between per-patient mean symptom severity and continuous sociodemographic variables.

Symptom	Dialysis vintage (months)	Household density (persons/room)
Pain	+0.165 (0.791)	-0.011 (0.976)
Fatigue	-0.062 (0.976)	+0.098 (0.877)
Nausea	-0.076 (0.975)	-0.022 (0.976)
Sleep disturbance	-0.015 (0.976)	-0.136 (0.779)
Worry / distress	-0.132 (0.869)	-0.049 (0.976)
Dyspnea	-0.090 (0.970)	-0.233 (0.584)
Memory impairment	+0.039 (0.976)	+0.135 (0.779)
Decreased appetite	+0.154 (0.825)	+0.055 (0.975)
Drowsiness	-0.005 (0.987)	-0.138 (0.779)
Dry mouth	+0.067 (0.976)	-0.030 (0.976)
Sadness	-0.018 (0.976)	-0.095 (0.887)
Vomiting	+0.012 (0.976)	-0.219 (0.584)
Numbness / tingling	-0.072 (0.975)	-0.173 (0.649)
Constipation	+0.006 (0.986)	-0.205 (0.584)
Diarrhea	-0.053 (0.976)	-0.263 (0.530)
Muscle cramps	-0.026 (0.976)	+0.020 (0.976)
Leg edema	+0.074 (0.975)	-0.039 (0.976)
Daydreaming / inattention	-0.014 (0.976)	+0.066 (0.970)
Restless legs	+0.136 (0.862)	-0.256 (0.530)
Cough	+0.163 (0.791)	-0.295 (0.442)

Symptom	Dialysis vintage (months)	Household density (persons/room)
Poor concentration	+0.208<br>(0.701)	+0.106<br>(0.862)
Dry skin	+0.093<br>(0.970)	-0.407<br>(0.099)
Pruritus	+0.010<br>(0.981)	-0.214<br>(0.584)
Overall health perception	+0.070<br>(0.975)	-0.156<br>(0.715)

Values shown as ρ (FDR-adjusted p-value). No association reached statistical significance after Benjamini–Hochberg correction (all padj > 0.05). n = 42 for dialysis vintage (some records missing); n = 69 for household density.
