# Supplementary material for: Symptom Clusters and Longitudinal Progression in Chronic Hemodialysis Patients: A Prospective Single-Center Study
Source: Healthcare (Basel). 2026 May 18;14(10):1375. doi: 10.3390/healthcare14101375 (PMC13205381; doi:10.3390/healthcare14101375)
Supplement: Supplementary file 1 [file healthcare-14-01375-s001.zip › Supplementary Table S4 - Kendall Correlation Matrix.pdf]

Supplementary Table S4. Kendall τb correlation coefficients between per-patient mean symptom severity and ordinal sociodemographic variables.

Symptom	Treatment frequency (per week)	Religiosity	Education level	Occupational status	Financial satisfaction	Housing satisfaction
Pain	+0.182 (0.584)	+0.008 (0.976)	+0.016 (0.976)	+0.049 (0.975)	-0.058 (0.934)	-0.083 (0.845)
Fatigue	+0.169 (0.584)	+0.091 (0.845)	-0.083 (0.862)	+0.046 (0.975)	+0.067 (0.900)	+0.073 (0.869)
Nausea	+0.140 (0.684)	-0.048 (0.975)	-0.013 (0.976)	+0.030 (0.976)	-0.032 (0.976)	-0.142 (0.584)
Sleep disturbance	+0.163 (0.584)	+0.141 (0.607)	-0.031 (0.976)	+0.174 (0.584)	-0.010 (0.976)	-0.013 (0.976)
Worry / distress	+0.176 (0.584)	+0.026 (0.976)	-0.107 (0.779)	-0.100 (0.791)	-0.109 (0.755)	-0.142 (0.584)
Dyspnea	+0.224 (0.530)	+0.100 (0.791)	-0.079 (0.869)	+0.049 (0.975)	+0.183 (0.547)	+0.149 (0.584)
Memory impairment	+0.094 (0.845)	-0.075 (0.887)	+0.038 (0.976)	+0.180 (0.584)	+0.115 (0.715)	+0.027 (0.976)
Decreased appetite	+0.138 (0.692)	-0.023 (0.976)	-0.120 (0.755)	+0.064 (0.934)	-0.007 (0.976)	-0.057 (0.934)
Drowsiness	+0.134 (0.701)	+0.035 (0.976)	-0.022 (0.976)	+0.085 (0.857)	+0.221 (0.442)	+0.115 (0.715)
Dry mouth	+0.108 (0.791)	+0.099 (0.791)	-0.095 (0.817)	+0.114 (0.755)	+0.150 (0.584)	+0.023 (0.976)
Sadness	+0.219 (0.530)	-0.025 (0.976)	-0.097 (0.808)	+0.150 (0.584)	+0.029 (0.976)	-0.062 (0.934)
Vomiting	+0.219 (0.530)	-0.005 (0.984)	-0.053 (0.970)	-0.018 (0.976)	-0.038 (0.976)	-0.082 (0.862)
Numbness / tingling	+0.176 (0.584)	+0.115 (0.760)	-0.064 (0.934)	+0.088 (0.854)	+0.041 (0.975)	-0.066 (0.906)
Constipation	+0.066	+0.209	-0.062	+0.114	+0.103	+0.018

Symptom	Treatment frequency (per week)	Religiosity	Education level	Occupational status	Financial satisfaction	Housing satisfaction
	(0.934)	(0.530)	(0.934)	(0.755)	(0.779)	(0.976)
Diarrhea	+0.156<br>(0.592)	-0.019<br>(0.976)	-0.017<br>(0.976)	-0.015<br>(0.976)	+0.140<br>(0.584)	+0.187<br>(0.538)
Muscle cramps	+0.048<br>(0.975)	+0.117<br>(0.755)	-0.067<br>(0.933)	+0.132<br>(0.659)	+0.050<br>(0.970)	+0.008<br>(0.976)
Leg edema	-0.045<br>(0.975)	+0.059<br>(0.958)	-0.022<br>(0.976)	-0.034<br>(0.976)	+0.043<br>(0.975)	-0.021<br>(0.976)
Daydreaming / inattention	+0.055<br>(0.970)	-0.024<br>(0.976)	-0.062<br>(0.934)	+0.037<br>(0.976)	+0.078<br>(0.862)	-0.011<br>(0.976)
Restless legs	+0.175<br>(0.584)	+0.074<br>(0.892)	+0.000<br>(1.000)	+0.154<br>(0.584)	+0.141<br>(0.584)	+0.101<br>(0.779)
Cough	+0.176<br>(0.584)	+0.145<br>(0.607)	-0.077<br>(0.877)	+0.099<br>(0.791)	+0.270<br>(0.174)	+0.093<br>(0.791)
Poor concentration	+0.023<br>(0.976)	-0.079<br>(0.869)	-0.055<br>(0.970)	+0.012<br>(0.976)	+0.135<br>(0.607)	-0.008<br>(0.976)
Dry skin	+0.057<br>(0.970)	+0.153<br>(0.584)	+0.018<br>(0.976)	+0.030<br>(0.976)	+0.260<br>(0.174)	+0.286<br>(0.126)
Pruritus	-0.041<br>(0.976)	+0.151<br>(0.584)	-0.015<br>(0.976)	+0.016<br>(0.976)	+0.166<br>(0.584)	+0.203<br>(0.530)
Overall health perception	+0.000<br>(1.000)	+0.014<br>(0.976)	+0.183<br>(0.584)	-0.067<br>(0.934)	-0.004<br>(0.986)	+0.140<br>(0.607)

Values shown as τb (FDR-adjusted p-value). No association reached statistical significance after Benjamini–Hochberg correction (all padj > 0.05). n = 69 for all variables.
