# Supplementary material for: Symptom Clusters and Longitudinal Progression in Chronic Hemodialysis Patients: A Prospective Single-Center Study
Source: Healthcare (Basel). 2026 May 18;14(10):1375. doi: 10.3390/healthcare14101375 (PMC13205381; doi:10.3390/healthcare14101375)
Supplement: Supplementary file 1 [file healthcare-14-01375-s001.zip › Supplementary Table S5 - DM-adjusted Mixed Models.pdf]

Supplementary Table S5. Sensitivity analysis with DM-adjusted linear mixed-effects models for cluster symptom trajectories

Cluster	Model	N	Observations	β (time)	SE	95% CI	p (time)	β (DM)	p (DM)
Emotional-Physical	Unadjusted	46	121	−0.154	0.353	−0.847 to 0.539	0.663		
	DM-adjusted			−0.142	0.354	−0.835 to 0.551	0.688	0.488	0.254
Fatigue-Sleep	Unadjusted	46	121	0.049	0.341	−0.620 to 0.718	0.886		
	DM-adjusted			0.047	0.342	−0.623 to 0.717	0.891	−0.035	0.937
Gastrointestinal	Unadjusted	46	121	**−1.057**	0.311	−1.667 to −0.447	**<0.001**		
	DM-adjusted			**−1.031**	0.310	−1.639 to −0.423	**<0.001**	**0.712**	**0.032**
Neurological / Lower-limb	Unadjusted	46	121	−0.004	0.436	−0.858 to 0.850	0.993		
	DM-adjusted			−0.009	0.437	−0.865 to 0.847	0.983	−0.138	0.751
Sensory-Skin	Unadjusted	46	121	0.261	0.397	−0.518 to 1.039	0.512		
	DM-adjusted			0.269	0.397	−0.510 to 1.048	0.498	0.289	0.535

Model: cluster_score ~ time + DM + (1 | patient), REML. β (time) = change per year in mean symptom score (0–10 scale); β (DM) = difference in cluster score for DM vs. No DM patients; SE = standard error; CI = 95% confidence interval. Bold values indicate p < 0.05. N = number of patients with ≥2 timepoints; Observations = total patient-visit records. Primary unadjusted results are reported in the main text.
