# Supplementary figures and images for: Symptom Clusters and Longitudinal Progression in Chronic Hemodialysis Patients: A Prospective Single-Center Study
Source: Healthcare (Basel). 2026 May 18;14(10):1375. doi: 10.3390/healthcare14101375 (PMC13205381; doi:10.3390/healthcare14101375)

# Elbow Plot for Optimal Number of Symptom Clusters

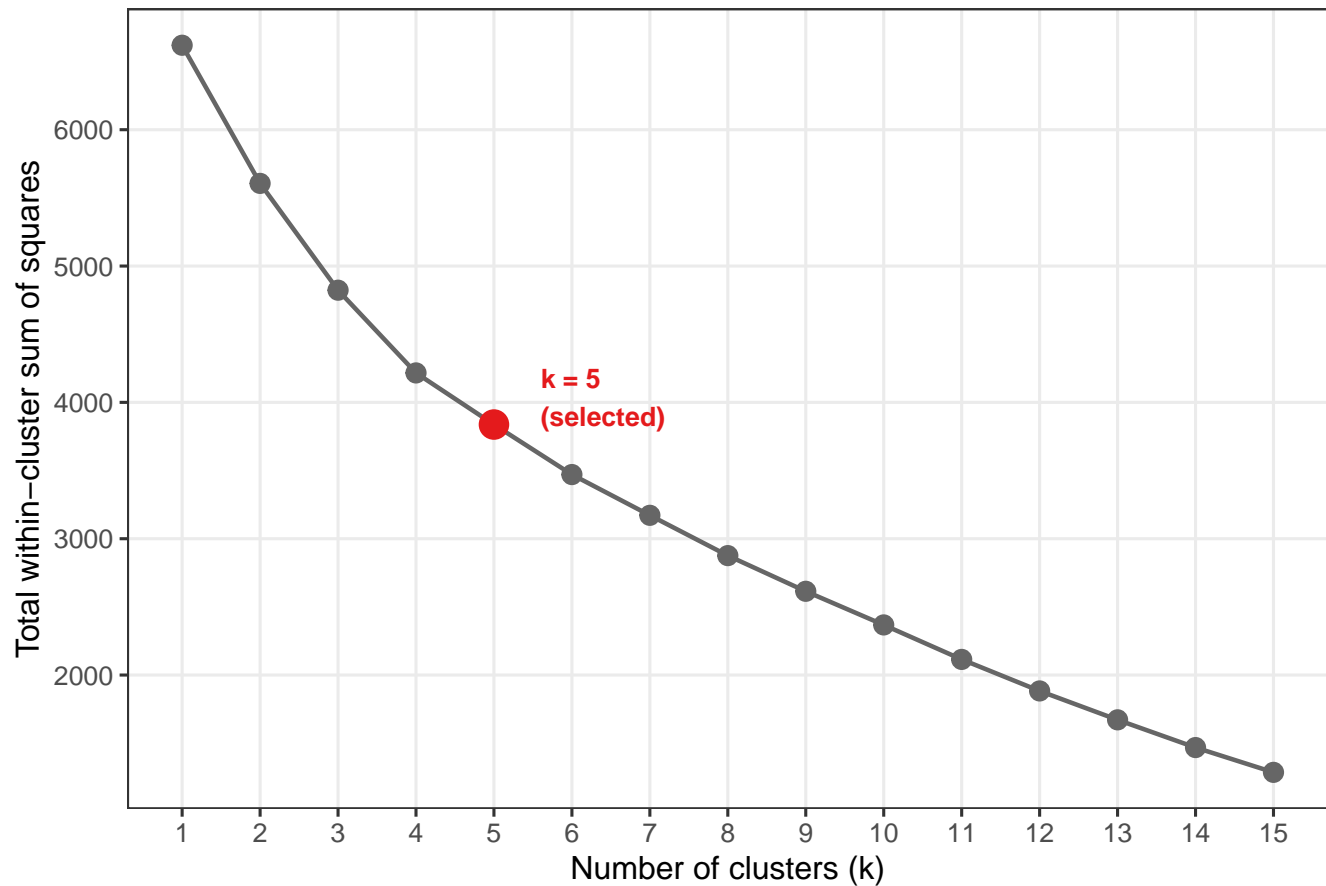

Supplement: Supplementary file 1 [file healthcare-14-01375-s001.zip › Supplementary Figure S1 - Cluster Elbow Plot.pdf]

A T1 (Baseline)

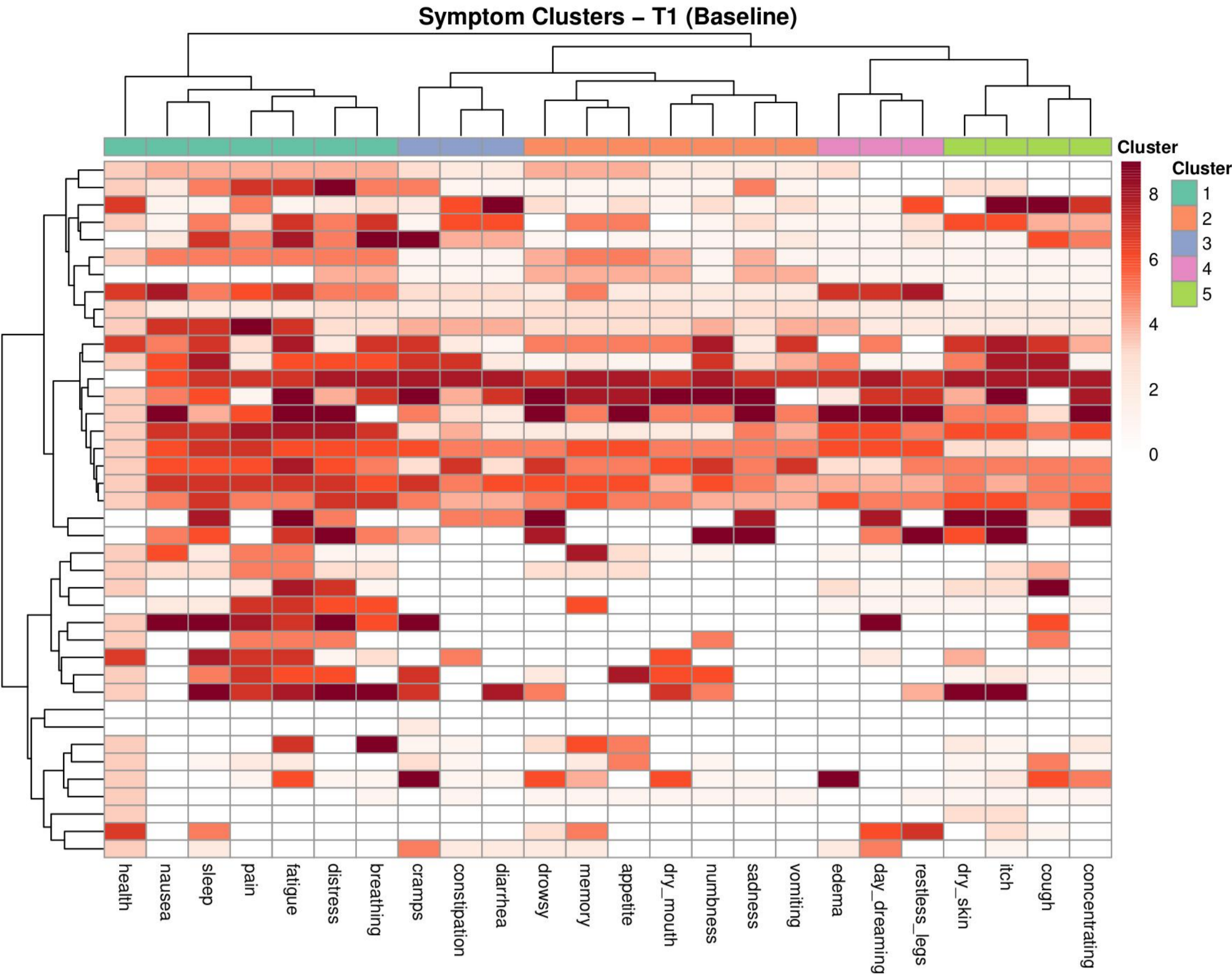

B T2 (~6 months)

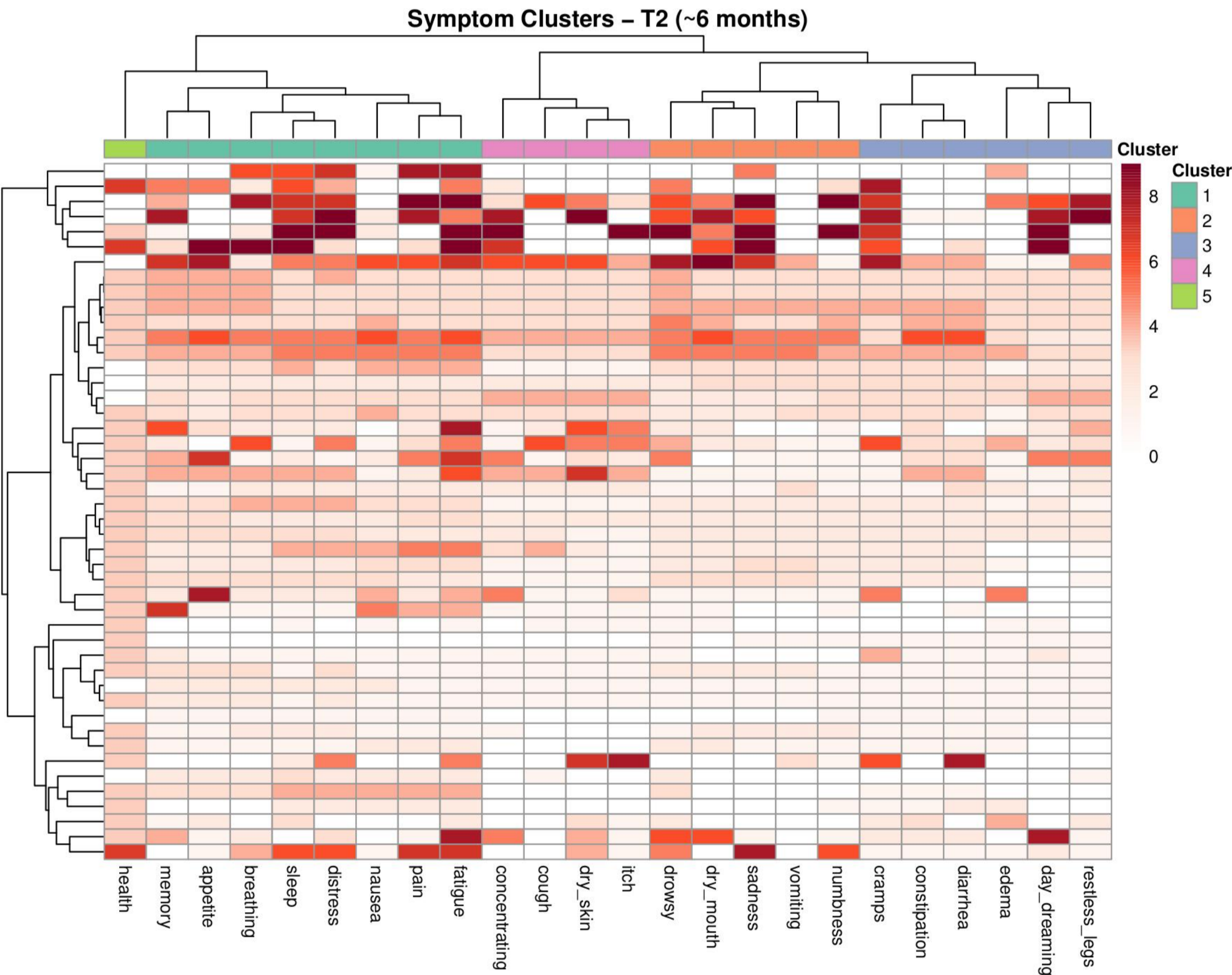

C T3 (~12 months)

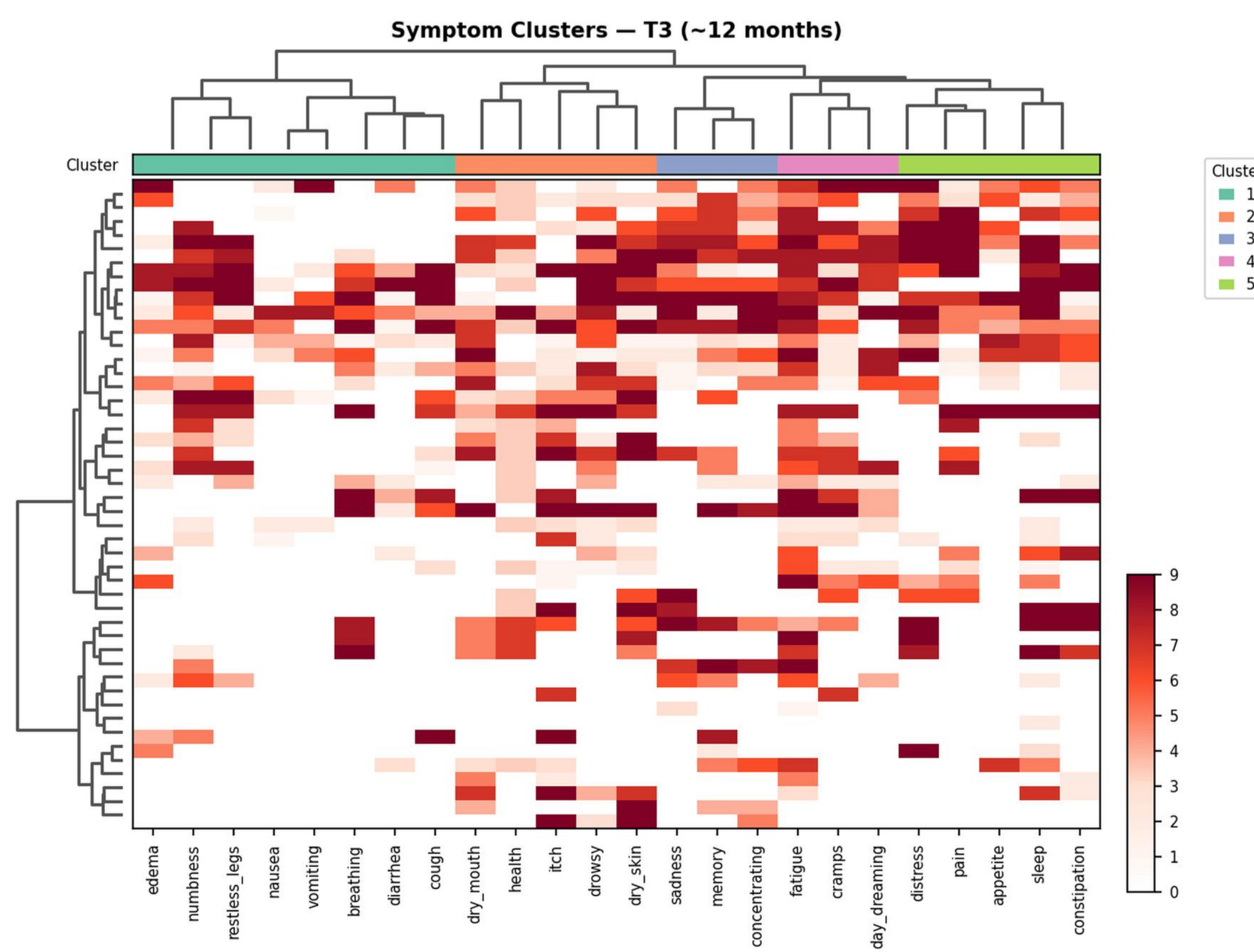

Supplement: Supplementary file 1 [file healthcare-14-01375-s001.zip › Supplementary Figure S3 - Cluster Stability Heatmaps T1 T2 T3.pdf]

# IDS on GDM2 distance – T1 (Baseline)

GDM2 distance + classical MDS | n = 45 patients

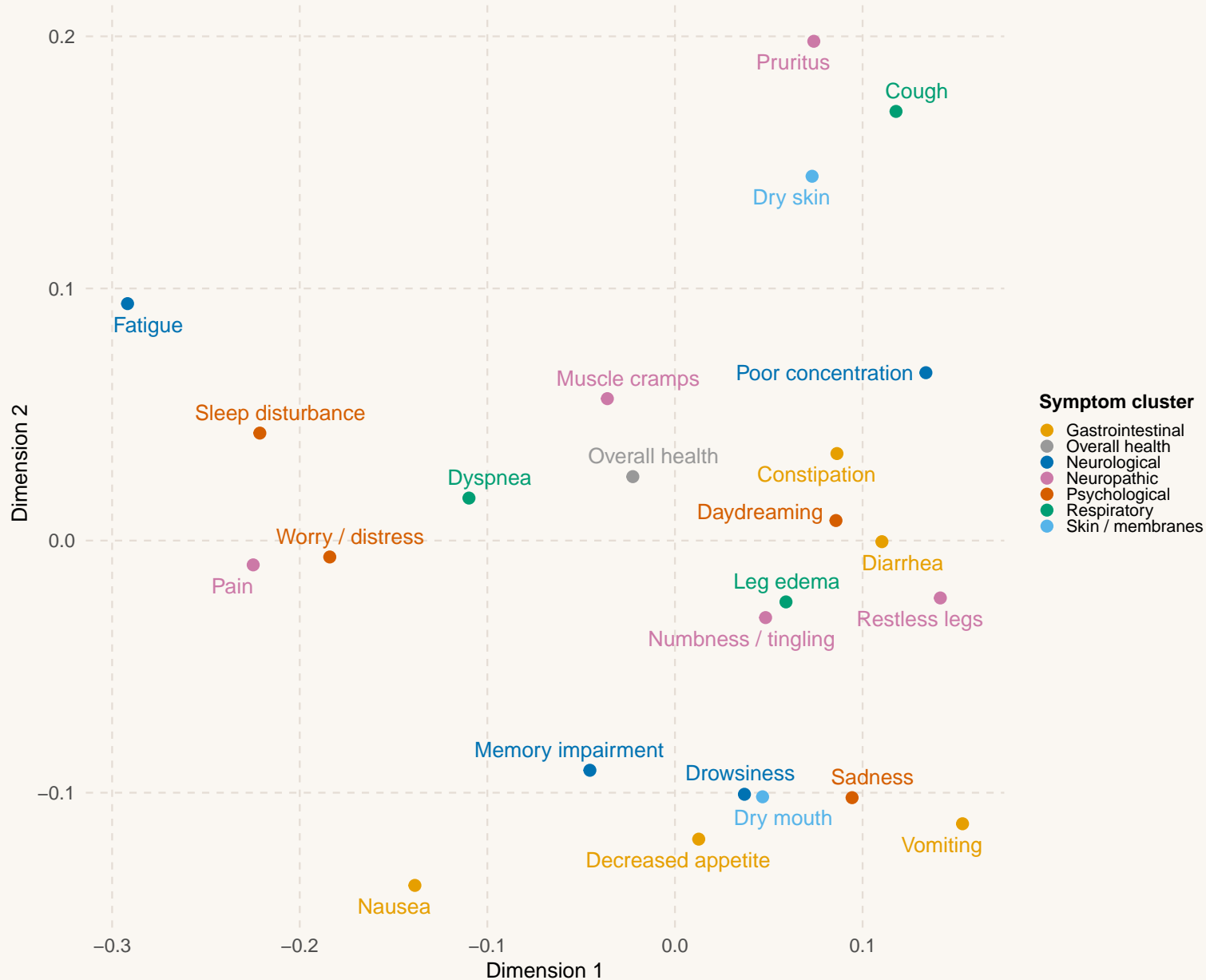

Supplement: Supplementary file 1 [file healthcare-14-01375-s001.zip › Supplementary Figure S4a - GDM2 Cluster Stability T1 Baseline.pdf]

# IDS on GDM2 distance – T2 (~6 months)

GDM2 distance + classical MDS | n = 53 patients

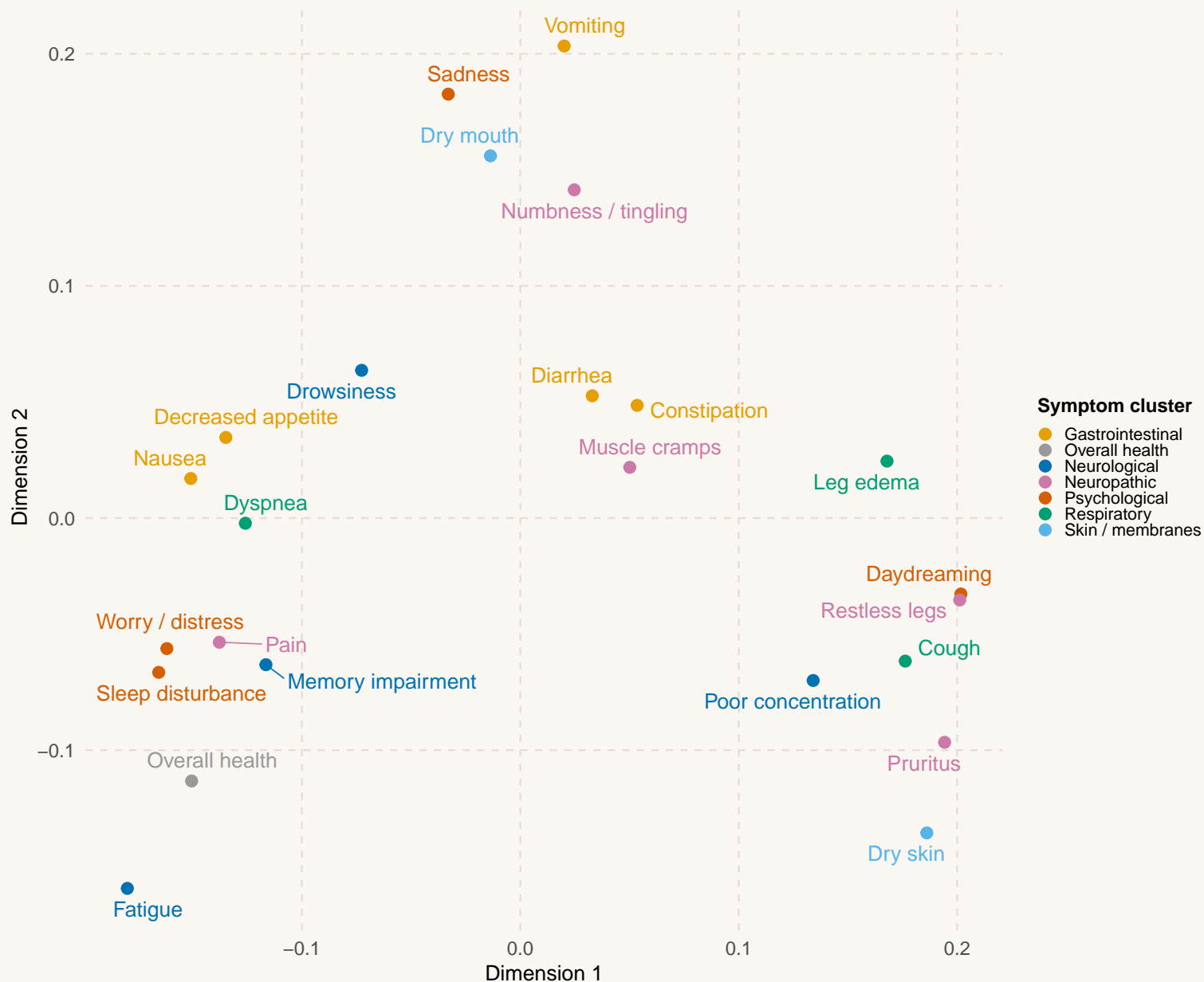

Supplement: Supplementary file 1 [file healthcare-14-01375-s001.zip › Supplementary Figure S4b - GDM2 Cluster Stability T2 6months.pdf]

# IDS on GDM2 distance – T3 (~12 months)

GDM2 distance + classical MDS | n = 46 patients

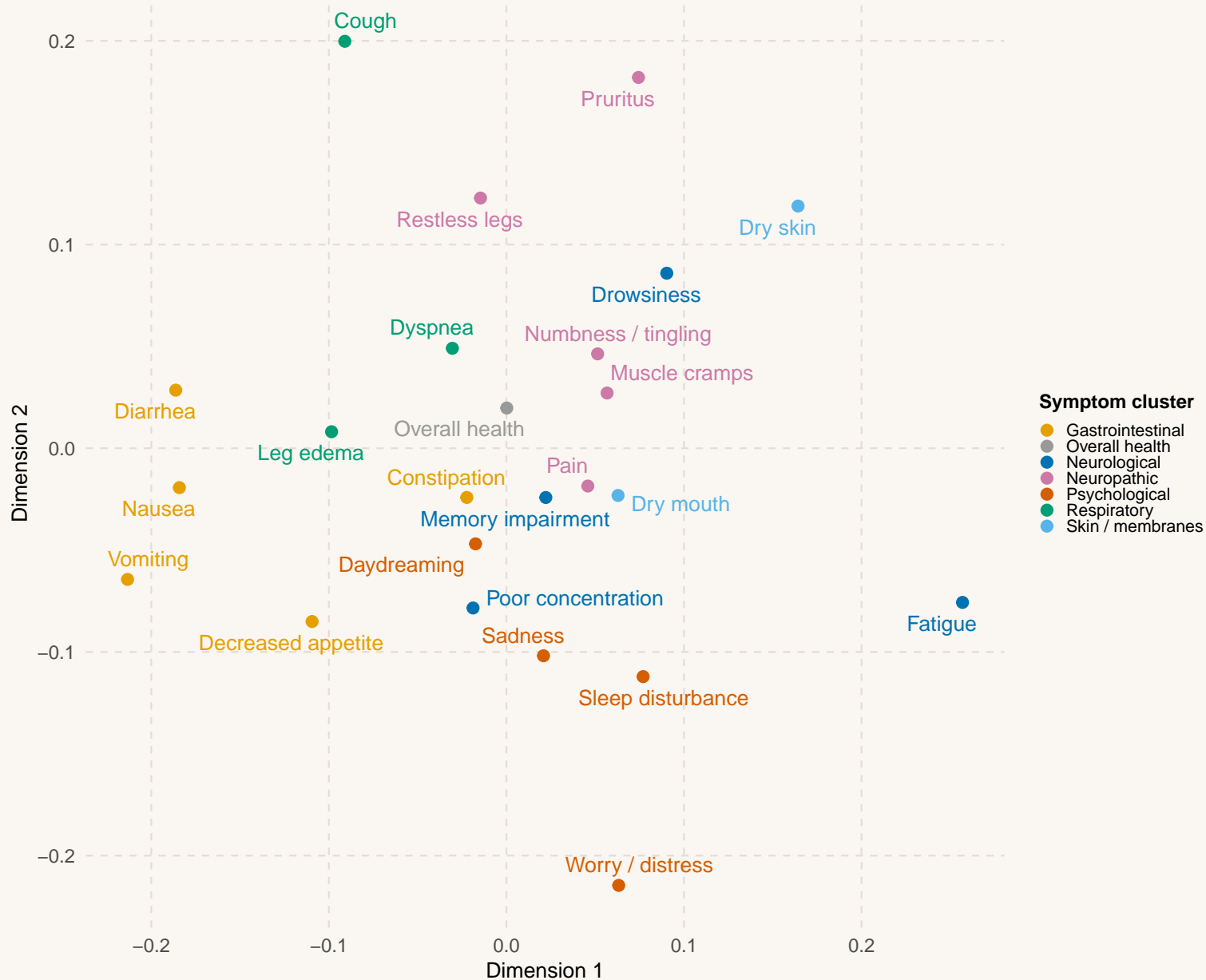

Supplement: Supplementary file 1 [file healthcare-14-01375-s001.zip › Supplementary Figure S4c - GDM2 Cluster Stability T3 12months.pdf]

# IDS on GDM2 distance – Mean across T1–T3

GDM2 distance + classical MDS | n = 69 patients

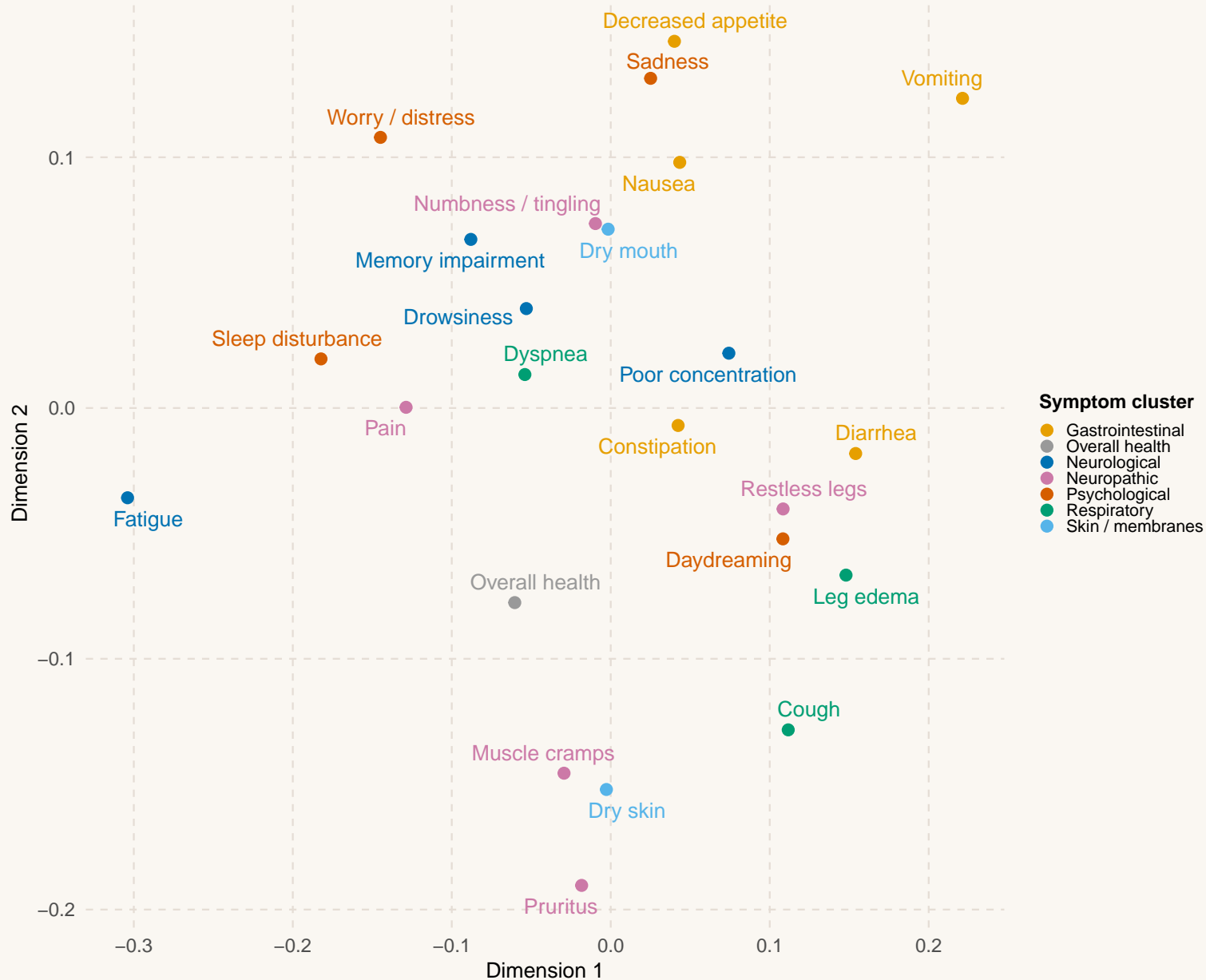

Supplement: Supplementary file 1 [file healthcare-14-01375-s001.zip › Supplementary Figure S4d - GDM2 Cluster Stability Mean.pdf]

# Per-symptom Silhouette Widths at k = 5

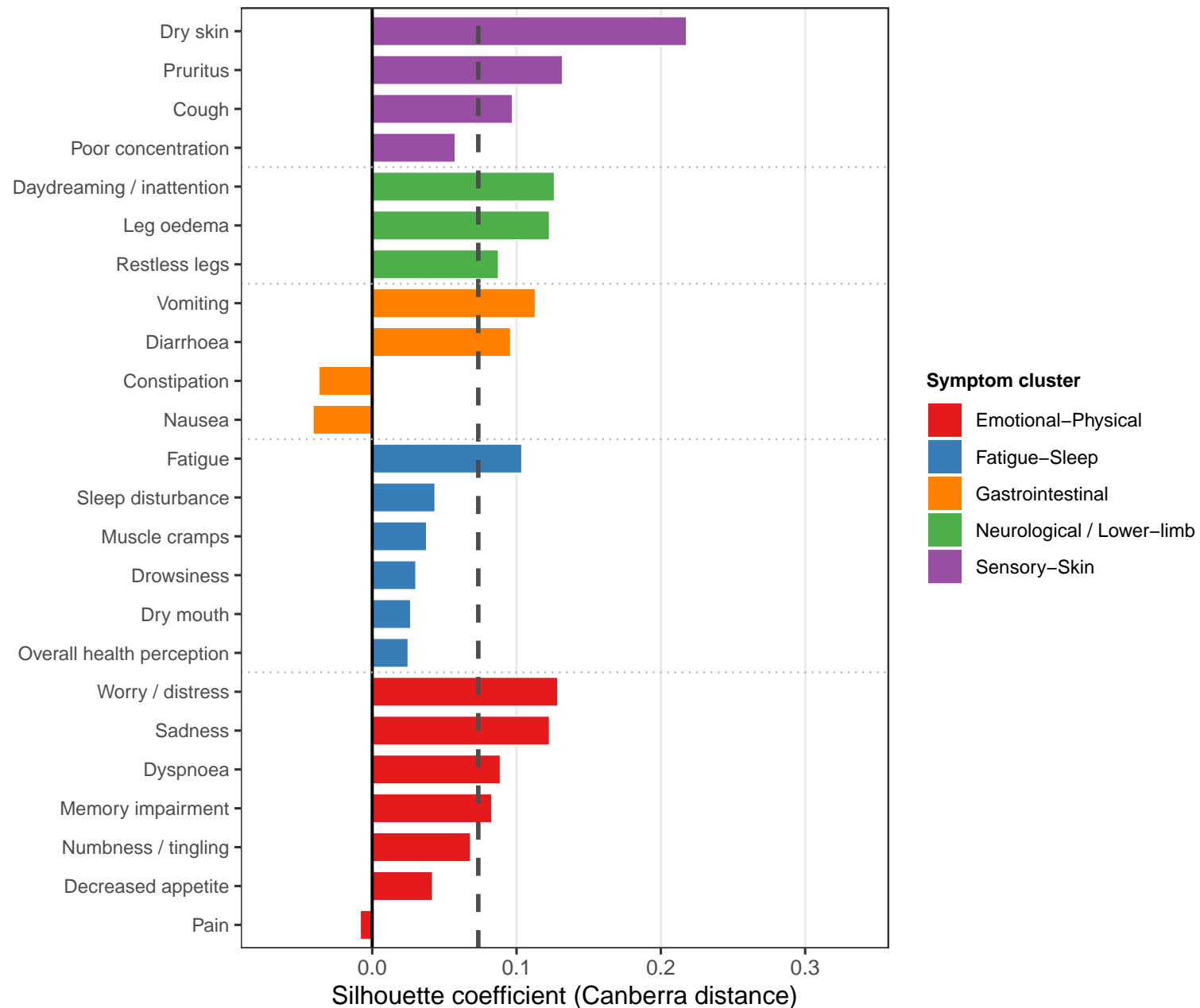

Supplement: Supplementary file 1 [file healthcare-14-01375-s001.zip › Supplementary Figure S6 - Silhouette_k5.pdf]
